# IMP^2^ART systematic review of education for healthcare professionals implementing supported self-management for asthma

**DOI:** 10.1038/s41533-018-0108-4

**Published:** 2018-11-06

**Authors:** Nicola McCleary, Amanda Andrews, Audrey Buelo, Mireille Captieux, Susan Morrow, Sharon Wiener-Ogilvie, Monica Fletcher, Liz Steed, Stephanie J. C. Taylor, Hilary Pinnock

**Affiliations:** 1 Asthma UK Centre for Applied Research, Usher Institute of Population Health Sciences and Informatics, University of Edinburgh, Edinburgh, UK; 2 Clinical Epidemiology Program, Ottawa Hospital Research Institute, Ottawa, Canada; 3 School of Epidemiology and Public Health, University of Ottawa, Ottawa, Canada; 4 Education for Health, Warwick, UK; 5 Usher Institute of Population Health Sciences and Informatics, University of Edinburgh, Edinburgh, UK; 6 Healthcare Improvement Scotland, Edinburgh, UK; 7 Multidisciplinary Evidence Synthesis Hub (mEsh), Centre for Primary Care and Public Health, Barts and The London School of Medicine and Dentistry, Queen Mary University of London, London, UK

## Abstract

Despite a robust evidence base for its effectiveness, implementation of supported self-management for asthma is suboptimal. Professional education is an implementation strategy with proven effectiveness, though the specific features linked with effectiveness are often unclear. We performed a systematic review of randomised controlled trials and controlled clinical trials (published from 1990 and updated to May 2017 using forward citation searching) to determine the effectiveness of professional education on asthma self-management support and identify features of effective initiatives. Primary outcomes reflected professional behaviour change (provision of asthma action plans) and patient outcomes (asthma control; unscheduled care). Data were coded using the Effective Practice and Organisation of Care Taxonomy, the Theoretical Domains Framework (TDF), and Bloom’s Taxonomy and synthesised narratively. Of 15,637 articles identified, 18 (reporting 15 studies including 21 educational initiatives) met inclusion criteria. Risk of bias was high for five studies, and unclear for 10. Three of 6 initiatives improved action plan provision; 1/2 improved asthma control; and 2/7 reduced unscheduled care. Compared to ineffective initiatives, effective initiatives were more often coded as being guideline-based; involving local opinion leaders; including inter-professional education; and addressing the TDF domains ‘social influences’; ‘environmental context and resources’; ‘behavioural regulation’; ‘beliefs about consequences’; and ‘social/professional role and identity’. Findings should be interpreted cautiously as many strategies were specified infrequently. However, identified features warrant further investigation as part of implementation strategies aiming to improve the provision of supported self-management for asthma.

## Introduction

Between 2005 and 2015, the global prevalence of asthma increased by 9.5%, with a corresponding increase in the years lived with disability of 9.4%.^1^ Since 1990, clinical guidelines have recommended that people with asthma be supported to manage their own condition.^2–4^ Supported self-management can be defined as *“providing information and encouragement to help people maintain greater control by understanding their condition and being able to monitor and take appropriate action”*.^5^ The Practical Systematic Review of Self-Management Support (PRISMS) meta-review for 14 long-term conditions (LTCs) confirmed the importance of supported self-management as a key component of high-quality care for people with LTCs.^6^ In the context of asthma, this work identified 23 systematic reviews synthesising data from 261 unique randomised controlled trials (RCTs) and concluded that self-management education for patients, reinforced by a written personalized asthma action plan (PAAP) and supported by regular review with a health care professional, reduces unscheduled care and improves markers of asthma control and quality of life.^6,7^

Although the evidence for the effectiveness of supported self-management in asthma is robust, implementation remains poor in routine clinical practice. An Asthma UK survey estimated that only 24% of people with asthma currently have a PAAP,^8^ and the National Review of Asthma Deaths identified inadequate routine care, including lack of self-management education reinforced with a PAAP, as a factor in 62% of the deaths investigated.^9^ Further understanding of implementation in this area is therefore essential. The key conclusion drawn based on the PRISMS work was that integration of supported self-management for asthma into routine practice requires a whole-systems implementation strategy in which motivated, skilled professionals support motivated, informed patients within an organisation that values, promotes and monitors supported self-management.^10^

We have therefore undertaken preliminary work to inform the development of such an implementation strategy. Because the provision of self-management support requires healthcare professionals to change their clinical behaviour, professionals whose role is to support patients are important targets for strategies aiming to improve the implementation of self-management support. As a component of an implementation strategy, professional education can address knowledge regarding the concept of supported self-management, and associated skills to support this change of behaviour in the professional.^6^ Cochrane reviews have shown that educational meetings/workshops, educational outreach visits, and printed educational materials can positively influence outcomes related to professional behaviour,^11–13^ while inter-professional education can improve clinical care.^14^ Previous asthma-related reviews have shown that professional education is an important component of implementation strategies focusing on supported self-management for asthma, and is associated with improvements in the process of care.^10,15^ However, professional education is a complex intervention and as such educational initiatives are likely to be composed of multiple parts. Additionally, specific features may differ between initiatives described as ‘professional education’. For example; there may be differences in the strategies involved (e.g. out-of-office educational meetings vs. educational outreach visits at practices); whom the initiative is directed at (e.g. individual professionals vs. practice teams); barriers to change targeted (e.g. knowledge/skills vs. confidence vs. access to resources), as well as the proposed active content of the education. The specific features of educational initiatives linked with effectiveness, and the barriers addressed by those features, are still unclear.

In the current study, we therefore aimed to synthesise the evidence regarding educational initiatives for professionals involved in self-management support for asthma. The objectives were to: (i) assess the effectiveness of the initiatives in changing professional behaviour and improving patient outcomes; and (ii) identify features of effective initiatives by mapping the strategies used to deliver the initiatives, the barriers targeted for change, and the educational goals.

## Results

### Study selection

**Fig. 1:**
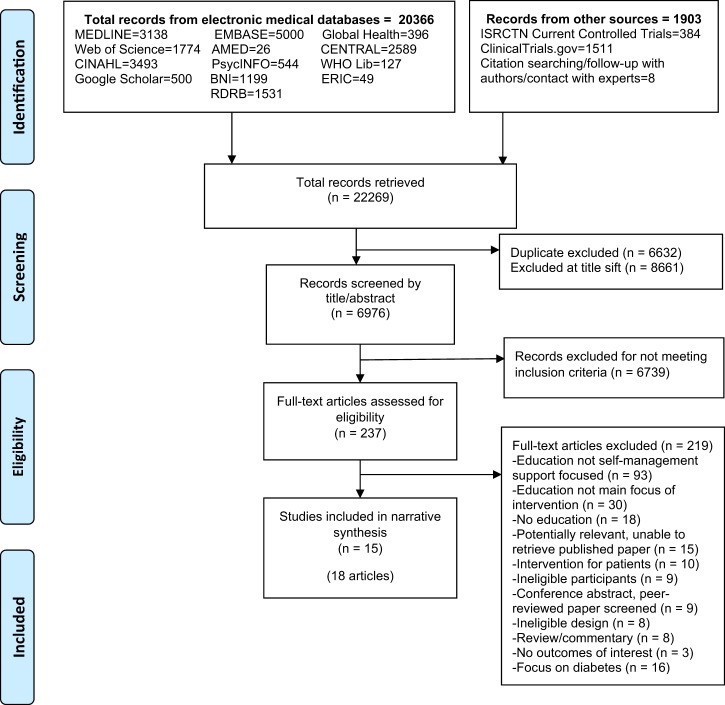
PRISMA flow diagram for database search of studies evaluating education for professionals implementing supported self-management for asthma. Notes: For Google Scholar, the first 500 hits were selected for screening. Searches included interventions reporting educational initiatives related to asthma and diabetes; studies separated at full text screening and synthesised separately

A total of 15,637 records were retrieved (after de-duplication), of which 18 articles^16–33^ reporting 15 studies were included in the review (Fig. 1).

### Characteristics of studies

Characteristics of the included studies are summarised in Table 1. Studies were published between 1993 and 2016 and took place across seven countries: USA (6),^16–21,24,26,29^ Australia (2),^28,31^ Israel (2),^23,33^ UK (2),^22,25^ the Netherlands (1),^30^ Singapore (1),^27^ and Sweden (1).^32^ Eight were cluster RCTs,^17–19,22,24–26,29,30^ three were RCTs,^16,20,21,27,28^ and four were CCTs.^23,31–33^ Three comprised education of primary care physicians only,^17,28,32^ two of paediatricians,^16,18–21^ two of nurses,^22,27^ and the remaining eight comprised education of more than one professional group (e.g. nurses and physicians).^23–26,29–31,33^

**Table 1:** Characteristics of included studies and key implementation and health outcomes

Study, year, country, design, risk of bias, duration	Setting, participants recruited	Brief intervention description, control	Key results BOLD are significant results * indicate the trial’s defined primary outcomes
Studies which evaluated the PACE programme			
Bruzzese et al.^17^ 2006 USA 2-group cluster RCT Unclear RoB 2 yr	44 schools = clusters PCPs: NR 591 low-income ethnic minority families (307 I, 284 C)	PACE initiative: develops skills for treating asthma, including supporting patients & families to self-manage Incentives: CME credits and catered meals; Invitation letter signed by Commissioner of Health *Control:* Standard care	*Implementation outcome:* NR *Health outcome:* Compared to control, at 2 yrs difference in patient reported (Mean/yr (SD): • Urgent physician visits: I: 1.7 (3.0), C: 1.8 (3.6) *p* = NS • ED visits: I: 0.9 (2.2), C: 0.9 (1.8) *p* = NS • Hospitalisations: I: 0.2 (0.6), C: 0.1 (0.3) *p* < .05 (favours C) Similar results at 1 yr
Cabana et al.^18,19^ 2006 USA 2-group cluster RCT Unclear RoB 2 yr	10 cities/ regions = clusters PC paediatricians practice: 94 (51 I, 43 C) Children/parents: 870 (418 I; 452 C)	PACE initiative: develops skills for treating asthma, including supporting patients & families to self-manage Incentives: CME credits, certificate, honorarium *Control:* Standard care (but received honorarium)	*Implementation outcome:* NR *Health outcome:* Compared to control, at 1 yr difference in change in patient reported (Mean change): • Urgent office visits: 1 yr: I: −1.07, C: −0.9, *p* = NS; • **ED visits: I: −0.55, C: −0.30**, ***p*** **<** **.05 (sustained at 2** **yr**, ***p*** **<** **0.038)** • Hospitalisations: I: −0.06, C: −0.06, ***p*** = NS
Clark et al.^16,20,21^ 1998 USA 2-group RCT Unclear RoB 2 yr	74 practices Paediatricians: 74 (38 I, 36 C) Children/parents: 637 (336 I, 301 C)	PACE initiative: develops skills for treating asthma, including supporting patients & families to self-manage *Control:* Standard care	*Implementation outcome:* Compared to control: • **At 22** **m more parents had written plan I: 26%, C: 16%, OR 1.74,** ***p*** **=** **.03** • **At 5** **m more physicians gave written plan I: 4.30, C: 3.46**, ***p*** **=** **0.001;** • *Health outcome:* Compared to control, at 2 yr there was (Mean): • No difference in patient reported ED visits: I: 0.29, C: 0.47, *p* = 0.44 • **Fewer patient reported hospitalisations: I 0.03, C 0.10**, ***p*** **=** **0.03**
Griffiths et al.^25^ 2016 UK 2-group cluster RCT Unclear RoB 1 yr	84 PCPs = clusters Nurses & GPs: NR South Asian adults/children: 375 (183 I, 192 C)	PACE initiative adapted for UK clinicians caring for South Asian patients: develops skills for treating asthma, including supporting patients & families to self-manage. Two lunchtime seminars *Control:* Standard care	*Implementation outcome:* NR *Health outcome:* Compared to control, at 1 yr no difference in: * Unscheduled care: OR 0.71 (95% CI 0.43 to 1.20), *p* = .20 * Time to first unscheduled care: HR 1.19 (95% CI 0.92 to 1.53) *p* = 0.19 Compared to control, at 3 m there was greater improvement in • **QoL (AQ20) mean diff −2.56 (95% CI −3.89 to −1.24)**, ***p*** **<** **0.001**
Shah et al.^28^ 2011 Australia 2-group RCT Unclear RoB 1 yr	109 PCPs GPs: 150 (78 I, 72 C) Children/parents: 221 (111 I, 110 C)	PACE initiative: adapted for Australian Cycle of Care develops skills for treating asthma, including supporting patients & families to self-manage *Control:* Standard care	*Implementation outcome:* Compared to control, at 1 yr more: • **Parents had a written action plan I: 61% C: 46% diff 15% (95% CI 2 to 28%)** ***p*** **=** **0.046** • **GPs provided written action plan** **>** **70% of the time I: 76% C: 53% diff 23% (95% CI 11 to 36%)** ***p*** **=** **0.002** *Health outcome:* Compared to control, at 1 yr there was no difference in: • Hospitalization: I: 18% C: 12% diff 6% (95% CI −4 to 15%) *p* = 0.12
Studies which evaluated initiatives other than PACE			
Cleland et al.^22^ 2007 UK 2-group cluster RCT Unclear RoB 6 months	13 PCPs = clusters (6 I, 7 C) Nurses: NR Adults: routine data: 629 373 I; 256 C questionnaire: 236 (130 I, 106 C)	One interactive seminar on effective communication, self-management education, and use of action plans *Control:* Standard care	*Implementation outcome:* NR *Health outcome:* Compared to control, at 6 m there was no difference in: • Steroid courses: I: 1.07 (1.04–1.10) C: 1.11 (1.07–1.45) *p* = 0.12 • Compared to control, at 6 m there was: * **Improved QoL (miniAQLQ) I: 6.49 (6.40–6.59) C: 6.33 (6.23–6.44)** ***p*** **=** **0.03** • No difference in asthma control (ACQ): I: 3.14 (3.06–3.23) C: 3.2 (3.10–3.30) *p* = 0.43
Cohen et al.^23^ 2014 Israel 5-group CCT High RoB 2 yrs	PCPs in 5 HMO divisions = ‘clusters’ GPs: 258 (45 I1, 35 I2, 21 I3, 36 I4, 121 C) Nurses: NR Patients > 12 yr: 1056 (54 I1, 219 I2, 106 I3, 171 I4, 506 C)	Focused on effective communication, self-management education for patient/families, and use of action plans I1: GPs: Patients with uncontrolled asthma identified & targeted I2: GPs: I1 plus workshop I3: GPs & nurses: I1 plus simulation training I4: GPs & nurses: I1 plus I2 plus I3 *Control:* Standard care	*Implementation outcome:* NR *Health outcome:* **Change in rate of uncontrolled asthma (based on inhaler purchase data) 1** **yr-baseline I1: 0.15 I2: 0.14 I3: 0.20 I4: 0.28** **C: 0.003** • **Diff I1-C: 0.15 z-score 3.27** ***p*** **<** **0.01** • **Diff I2-C: 0.13 z-score 5.67** ***p*** **<** **0.01** • **Diff I3-C: 0.19 z-score 5.00** ***p*** **<** **0.01** • **Diff I4-C: 0.26 z-score 7.97** ***p*** **<** **0.01** Similar results at 2 yrs
Evans et al.^24^ 1997 USA 2-group cluster RCT Unclear RoB 2 yr	Clusters = 2 panels of PCPs (11 I, 11 C) Professionals: 134 (80 I, 54 C) Children/parents: 358	Creating a Medical Home for Asthma; Focused on effective communication, self-management education for patient/families, and use of action plans *Control* Standard care (but received guidelines)	*Implementation outcome:* Compared to control, at 2 yr there was: • no difference in the proportion of patients given written plan by physician: I: 78% C: 76% (*p* = NS) or nurse I: 60% C: 53% *p* = NS • **increased proportion received education by physician I: 71% C: 58%** ***p*** **<** **0.01** but no difference by nurse I: 61% C: 44% *p* = NS *Health outcome*: No difference in proportion who received oral steroid course I: 5% C: 1% *p* = NS **Difference in proportion who received** • **any β-agonist I: 74% C: 52%** ***p*** **<** **.05** • **any inhaled anti-inflammatory I: 25% C: 2%** ***p*** **<** **.001**
Homer et al.^26^ 2005 USA 2-group cluster RCT High RoB 1 yr	43 practices = clusters (22 I, 21 C) 3-member team (physician, nurse, office staff): NR Children/parents: 631 (294 I, 337 C)	Learning collaborative project: participants identified performance gaps in their own practices’ asthma care, and learning was based on these *Control:* Standard care	*Implementation outcome:* Compared to control, at 1 yr there was no difference in: * proportion of patients given written management plan: I: 54% C: 41% *p* = NS *Health outcome:* Compared to control, at 1 yr there was no difference in: • Hospitalisation: I: 2% C: 4% *p* = NS • ED visit: I: 17% C: 22% *p* = NS • Asthma attack: I: 40% C: 36% *p* = NS
Prabhakaran et al.^27^ 2012 Singapore 3-group RCT Unclear RoB 3 months	1 tertiary hospital Enroled nurses: 162 (59 I1, 55 I2, 48 I3) No patients recruited	Education on general management of asthma, specifically including self-management support I1: Workshop with practical skills I2: As I1 except lectures in e-learning format I3: combination of I1 and I2 *Control:* Interventions compared, no standard care control	*Implementation outcome:* Knowledge (not assessed with validated tool) *Health outcomes:* NR
Sheikh et al.^29^ 2016 USA 2-group cluster RCT Unclear RoB 2 yr	10 PC paediatric practices = clusters (5 I, 5 C) Professionals: NR Patients: routine data: NR	Education of Asthma Leaders in each practice on general management of asthma, specifically including self-management support *Control:* delayed intervention	*Implementation outcome:* Compared to control, at 1 yr there was increased recording of: • **Asthma education: I: 56.1%, C: 19.5%**, ***p*** **≤** **0.05** • **Asthma action plan: I: 29%, C: 5.4%**, ***p*** **≤** **0.05** Compared to control, at 1 yr there was no difference in: • Acute care visit: I: 90.3%, C: 91.9%, NS
Smeele et al.^30^ 1999 Netherlands 2-group cluster RCT Unclear RoB 1 yr	PCPs in same ‘local group’ = cluster GPs: 34 practice assistants: NR Adult asthma or COPD patients: 544	Education covered general management of asthma (and COPD), specifically including self-management support *Control:* Standard care	*Implementation outcome:* Compared to control, at 1 yr there was no difference in change in proportion: • Patients receiving written education I: +3% C: +7% Diff −1% (−13 to 11%) *p* = 0.8 *Health outcome:* Compared to control, at 1 yr there was no difference in: • patients reporting exacerbations past 3 months (ratio) I: 0 C: −0.11 *p* = 0.1
Toelle et al.^31^ 1993 Australia 2-group CCT High RoB 9 months	Primary schools in 2 areas = ‘clusters’ HCPs: NR Children/families: 132 (72 I, 60 C)	Evening workshops and in-service education on effective communication, self-management education for patient/families, and use of action plans *Control*: Standard care	*Implementation outcome:* NR *Health outcome:* Compared to control, at 6 m there was no difference in: • Unscheduled doctor/ ED visits, mean (95% CI) I: 1.51 (0.94 to 2.08) C: 1.67 (1.01 to 2.33) *p* = NS • Symptoms limiting activity, % (95% CI) I: 18.60% (7.0% to 30.2%) C: 8.30% (0% to 17.3%) *p* = NS
Tomson et al.^32^ 1997 Sweden 2-group CCT High RoB 18 months	2 localities = ’clusters’ 30 PCPs (21 I, 9 C) GPs: NR Patients: 331 (249 I, 82 C)	Academic detailing for diagnosis and treatment of asthma, covering general management of asthma, specifically including self-management support *Control*: Standard care	*Implementation outcome:* Compared to control, at 1 yr there was no difference in proportion: • Given a PEF-based self-management plan I: 46% C: 32% *p* = 0.05 *Health outcome:* Compared to control, at 1 yr there was no difference in ratios of prescribed inhaled β-agonists to inhaled glucocorticoids measured as defined daily doses: *p* = NS for areas/’clusters’
Volovitz et al.^33^ 2003 Israel 4-group CCT High RoB 9 months	PCPs in two regions within a HMO GPs & paediatricians: NR Adults & children: NR	Education covered general management of asthma, specifically including self-management support I1: Asthma education programme; application of learning to future consultations monitored I2: I1 except follow-up not monitored *Control 1*: Standard care, patients in same region *Control 2*: Standard care, patients in different region	*Implementation outcome:* data NR in full so not extracted *Health outcome*: Compared to control, at 9 m patient reported: • **Improvement in shortness of breath I1: 64% I2: 39%** ***p*** **>** **0.005 (significant)**

ACQ, Asthma control questionnaire, AQ20, Airways Questionnaire 20, AQLQ, Asthma Quality of Life Questionnaire, CCT, controlled clinical trial; C, control group, CME, continuing medical education; COPD, chronic obstructive pulmonary disease, ED, emergency department, GP, general practitioner, HMO, health maintenance organization, I, intervention group, NR, not reported, PACE, Physician Asthma Care Education, PCP, primary care physician, PEF, peak expiratory flow, QoL, quality of life, RCT, randomised controlled trial, RoB, risk of bias

Three studies evaluated multiple initiatives (i.e. had more than two trial arms)^23,27,33^ so that the 15 studies evaluated 21 professional education initiatives. Five evaluated the Physician Asthma Care Education (PACE) initiative, which aims to develop skills for treating childhood asthma, including supporting families to self-manage.^16–21,25,28^ Seven initiatives focused on effective communication, self-management education for patient/families, and use of action plans;^22–24,31^ one also included simulation training.^23^ One initiative was a learning collaborative project, where participants identified performance gaps in their own practices’ asthma care, and learning was based on these.^26^ In the remaining eight initiatives, the education covered general management of asthma, with self-management support being a specific topic addressed.^27,29,30,32,33^

### Risk of bias

Overall, the risk of bias was rated for each study as either unclear (10 studies) or high (5 studies: four CCTs,^23,31–33^ and a cluster RCT^26^ which reported potential contamination between intervention and control practices). Summary and component-specific risk of bias ratings are provided in Online Supplementary File 1, along with risk of bias assessment notes for included studies detailing the rationale for the risk assessments made.

### Effectiveness of educational initiatives

For around half of the initiatives (10/21) there was some evidence of effectiveness in changing professional behaviour and/or patient outcomes as compared to control conditions (Table 1, Online Supplementary Files 2–3).^16,18–21,23–25,28,29^ Action plan provision/ownership was assessed for six initiatives, though it was not clear that this always meant a PAAP (for example, phrases such as *care plan* and *instructions on how to adjust medicine when symptoms change* were used). Three initiatives improved provision/ownership,^16,20,21,28,29^ and three did not.^24,26,32^ Four initiatives (assessed in one study) may have reduced rates of uncontrolled asthma (based on the proxy measure of inhaler purchase data);^23^ however this study was assessed as having a high risk of bias. One underpowered study showed no between-group difference in asthma control.^22^ Unscheduled care was measured for seven initiatives. Two were inconsistently effective in reducing unscheduled care (for example, reduced emergency department visits, but not hospitalisations).^16,18–21^ The remaining five were ineffective.^17,25,26,28,31^ Various secondary outcomes were measured: one initiative improved receipt of some (but not all) medications,^24^ and another improved quality of life and self-efficacy.^25^

### Features of effective initiatives

**Fig. 2:**
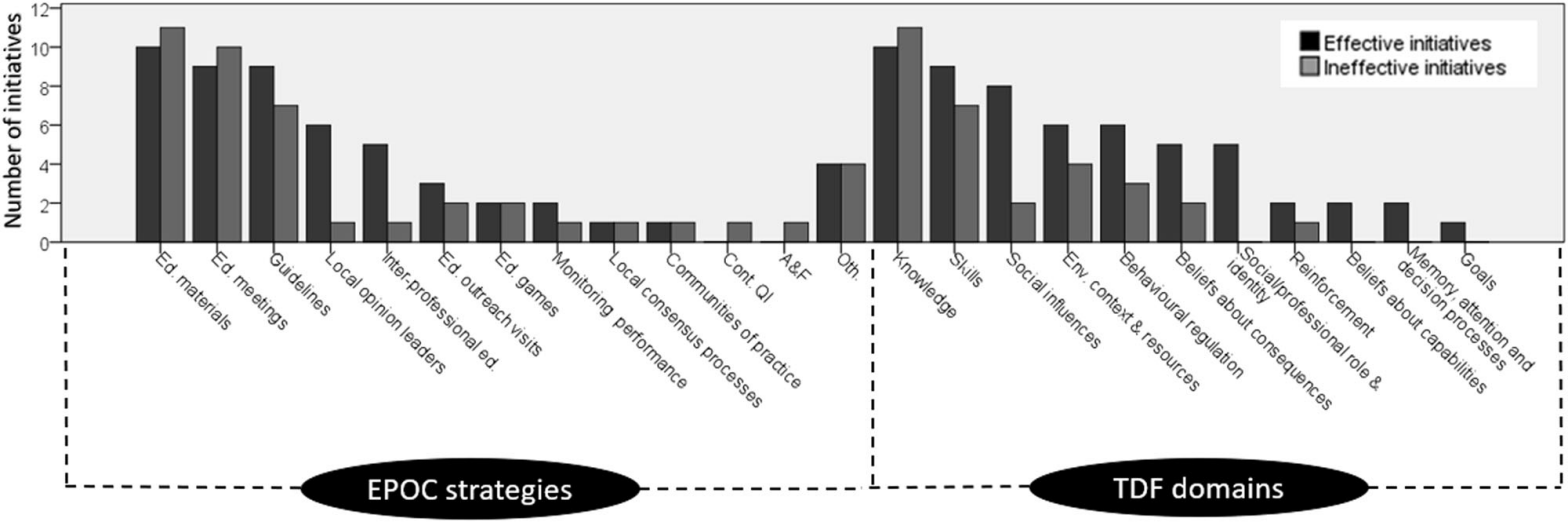
Cochrane EPOC taxonomy implementation strategies and TDF domains coded for effective compared to ineffective initiatives. Note: EPOC = Effective Practice and Organisation of Care; TDF = theoretical domains framework A&F = audit and feedback; Cont. QI = Continuous quality improvement; Ed. = education/educational; Env. = environmental; Oth. = other

The features of the educational initiatives are illustrated in Fig. 2, described below, and detailed in Tables 2–4.

**Table 2:** Cochrane EPOC taxonomy implementation strategies coded for asthma supported self-management educational initiatives

Study, country, risk of bias	Cabana 2006^a^ USA Unclear	Clark 1998^a^ USA Unclear	Griffiths 2016^a^ UK Unclear	Shah 2011^a^ Australia Unclear	Cohen 2014 Israel High				Evans 1997 USA Unclear	Sheikh 2016 USA Unclear	Bruzzese 2006^1^ USA Unclear	Cleland 2007 UK Unclear	Homer 2005 USA High	Prabhakaran 2012 Singapore Unclear			Smeele 1999 Netherlands Unclear	Toelle 1993 Australia High	Tomson 1997 Sweden High	Volovitz 2003 Israel High		
					I1	I2	I3	**I4**						I1	I2	I3				I1	I2	
Evidence of effectiveness?	Y	Y	Y	Y	Y	Y	Y	Y	Y	Y	N	N	N	N	N	N	N	N	N	N	N	
Implementation outcomes	NR	**Action plan**	NR	**Action plan**	NR				Action plan; **Patient education**	**Action plan; Patient education**	NR	NR	Action plan	Knowledge			Patient education	NR	Action plan	NR		
Health outcomes	Urgent office visit; **ED visit**; Hospital-isation	ED visit; **Hospital-isation**	Unscheduled care; Time to first unscheduled care; **QoL**	Hospital-isation	**Asthma control**				**Control medication**	Acute care visit	Urgent physician visit; ED visit; Hospital-isation	Steroid courses; **QoL**; Asthma control	ED visit; Hospital-isation; Asthma attacks	NR			Exacerbation	Unscheduled doctor/ ED visit; Symptoms limiting activity	Control medication	**Shortness of breath**		
Audit & feedback													✓									1
Monitoring the performance of the delivery of healthcare							✓	✓					✓									3
Communities of practice									✓				✓									2
Continuous quality improvement													✓									1
Educational meetings	✓	✓		✓	✓	✓	✓	✓	✓	✓	✓	✓	✓	✓	✓	✓	✓	✓		✓	✓	19
Educational materials	✓	✓	✓	✓	✓	✓	✓	✓	✓	✓	✓	✓	✓	✓	✓	✓	✓	✓	✓	✓	✓	21
Educational outreach visits/academic detailing			✓						✓	✓								✓	✓			5
Clinical practice guidelines	✓	✓	✓	✓		✓	✓	✓	✓	✓	✓	✓	✓				✓		✓	✓	✓	16
Inter-professional education			✓				✓	✓	✓	✓			✓									6
Local consensus processes									✓								✓					2
Local opinion leaders	✓	✓			✓	✓	✓	✓			✓											7
Educational games							✓	✓				✓					✓					4
Other^b^			✓			✓		✓		✓	✓							✓	✓	✓		8
Total	4	4	5	3	3	5	7	8	7	6	5	4	8	2	2	2	5	4	4	4	3	95

BOLD are significant results in favour of the intervention

Strategies not coded: Clinical incident reporting; Managerial supervision; Public release of performance data; Routine patient-reported outcome measures; Patient-mediated interventions; Tailored interventions; Reminders

EPOC: Effective Practice and Organisation of Care. I, intervention; N, no; NR, not reported; QoL, quality of life; Y, yes

^a^Study evaluated the Physician Asthma Care Education (PACE) programme

^b^Bruzzese: School health team training. Griffiths: patients received invitation to attend Chronic Disease Self Management Programme (CDSMP). Cohen: Training in the proper use of different kinds of inhalers. Sheikh: elements aimed at patients. Toelle: elements aimed at patients, parents, school teachers. Tomson: Generation of relevant topics for the information by direct contact with GPs before designing the information package. Volovitz: elements aimed at patients.

**Table 3:** TDF domains coded for asthma supported self-management educational initiatives

Study, country, risk of bias	Cabana 2006^1^ USA Unclear	Clark 1998^1^ USA Unclear	Griffiths 2016^1^ UK Unclear	Shah 2011^a^ Australia Unclear	Cohen 2014 Israel High				Evans 1997 USA Unclear	Sheikh 2016 USA Unclear	Bruzzese 2006^a^ USA Unclear	Cleland 2007 UK Unclear	Homer 2005 USA High	Prabhakaran 2012 Singapore Unclear			Smeele 1999 Netherlands Unclear	Toelle 1993 Australia High	Tomson 1997 Sweden High	Volovitz 2003 Israel High		
					I1	I2	I3	**I4**						I1	I2	I3				I1	I2	
Evidence of effectiveness?	Y	Y	Y	Y	Y	Y	Y	Y	Y	Y	N	N	N	N	N	N	N	N	N	N	N	
Implementation outcomes	NR	**Action plan**	NR	**Action plan**	NR				Action plan; **Patient education**	**Action plan; Patient education**	NR	NR	Action plan	Knowledge			Patient education	NR	Action plan	NR		
Health outcomes	Urgent office visit; **ED visit**; Hospital-isation	ED visit; **Hospital-isation**	Unscheduled care; Time to first unscheduled care; **QoL**	Hospital-isation	**Asthma control**				**Control medication**	Acute care visit	Urgent physician visit; ED visit; Hospital-isation	Steroid courses; **QoL**; Asthma control	ED visit; Hospital-isation; Asthma attacks	NR			Exacerbation	Unscheduled doctor/ED visit; Symptoms limiting activity	Control medication	**Shortness of breath**		
Knowledge	✓	✓	✓	✓	✓	✓	✓	✓	✓	✓	✓	✓	✓	✓	✓	✓	✓	✓	✓	✓	✓	21
Skills	✓	✓	✓	✓		✓	✓	✓	✓	✓	✓	✓	✓	✓	✓	✓	✓					16
Environmental context and resources	✓	✓	✓	✓					✓	✓	✓	✓	✓					✓				10
Belief about consequences	✓	✓	✓	✓					✓		✓	✓										7
Behavioural regulation	✓	✓	✓	✓			✓	✓			✓	✓	✓									9
Reinforcement	✓								✓		✓											3
Social influences	✓	✓	✓		✓	✓	✓	✓	✓		✓						✓					10
Belief about capabilities							✓	✓														2
Social/professional role and identity			✓				✓	✓	✓	✓												5
Goals									✓													1
Memory, attention, and decision processes							✓	✓														2
TOTAL	7	6	7	5	2	3	7	7	8	4	7	5	4	2	2	2	3	2	1	1	1	86

BOLD are significant results in favour of the intervention

Domains not coded: Optimism; Intentions; Emotion

TDF: Theoretical Domains Framework; I, intervention; N, no; Y, yes

^a^Study evaluated the Physician Asthma Care Education (PACE) programme

**Table 4:** Bloom’s Taxonomy levels coded for asthma supported self-management educational initiatives

Study, country, risk of bias	Cabana 2006^a^ USA Unclear	Clark 1998^a^ USA Unclear	Griffiths 2016^a^ UK Unclear	Shah 2011^a^ Australia Unclear	Cohen 2014 Israel High				Evans 1997 USA Unclear	Sheikh 2016 USA Unclear	Bruzzese 2006^a^ USA Unclear	Cleland 2007 UK Unclear	Homer 2005 USA High	Prabhakaran 2012 Singapore Unclear			Smeele 1999 Netherlands Unclear	Toelle 1993 Australia High	Tomson 1997 Sweden High	Volovitz 2003 Israel High	
					I1	I2	I3	**I4**						I1	I2	I3				I1	I2
Evidence of effectiveness?	Y	Y	Y	Y	Y	Y	Y	Y	Y	Y	N	N	N	N	N	N	N	N	N	N	N
Implementation outcomes	NR	**Action plan**	NR	**Action plan**	NR				Action plan; **Patient education**	**Action plan; Patient education**	NR	NR	Action plan	Knowledge			Patient education	NR	Action plan	NR	
Health outcomes	Urgent office visit; **ED visit**; Hospital-isation	ED visit; **Hospital-isation**	Unscheduled care; Time to first unscheduled care; **QoL**	Hospital-isation	**Asthma control**				**Control medication**	Acute care visit	Urgent physician visit; ED visit; Hospital-isation	Steroid courses; **QoL**; Asthma control	ED visit; Hospital-isation; Asthma attacks	NR			Exacerbation	Unscheduled doctor/ ED visit; Symptoms limiting activity	Control medication	**Shortness of breath**	
Low level thinking skills																					
Knowledge	✓	✓	✓	✓	✓	✓	✓	✓	✓	✓	✓	✓✓	✓	✓	✓	✓	✓	✓	✓	✓	✓
Comprehension	✓	✓	✓	✓			✓	✓	✓	✓	✓	✓	✓	✓	✓	✓	✓				
High level thinking skills																					
Application	✓	✓	✓	✓			✓	✓	✓	✓	✓	✓	✓	✓	✓	✓	✓				
Analysis							✓	✓	✓				✓								
Synthesis							✓	✓					✓								
Evaluation							✓	✓													

BOLD are significant results in favour of the intervention

I, intervention; N, no; Y, yes

^a^Study evaluated the Physician Asthma Care Education (PACE) programme

The EPOC implementation strategy ‘educational materials’ was coded for all 21 initiatives, and ‘educational meetings’ was coded for 19 of the 21. Compared to the ineffective initiatives, the 10 initiatives with some evidence of effectiveness more often included ‘clinical practice guidelines’, ‘local opinion leaders’, and ‘inter-professional education’. The TDF domain ‘knowledge’ was coded for all 21 initiatives, including both effective and ineffective initiatives. The domain ‘skills’ was coded for 16 of the 21 (9 of 10 effective; 7 of 11 ineffective). Nine further domains were coded at least once: five of these were coded for more than half of the initiatives with some evidence of effectiveness. These were ‘social influences’ (8 of 10 effective; 2 of 11 ineffective), ‘environmental context and resources’ (6 of 10 effective; 4 of 11 ineffective), ‘behavioural regulation’ (6 of 10 effective; 3 of 11 ineffective), ‘beliefs about consequences’ (5 of 10 effective; 2 of 11 ineffective), and ‘social/professional role and identity’ (5 of 10 effective; 0 of 11 ineffective). There appeared to be no clear differences in Bloom’s Taxonomy coding between effective compared to ineffective initiatives.

Tables 2–4 also include the key results from includes studies (specific outcomes and whether results were in favour of the intervention). There were no clear differences in coding between interventions which were effective for specific outcomes compared to those which were ineffective for those outcomes.

### Grey literature review

In total, 744 records were screened, and three studies were included.^34–36^ The studies were heterogeneous, and few methodological details were reported. Two studies had moderate or high risk of bias. Reports tended to highlight the positive, with all studies reporting some evidence of effectiveness, so we were unable to distinguish features of effective/ineffective initiatives. Two of the initiatives described involving stakeholders in the development of their initiative.^34,36^ Further details are provided in Online Supplementary File 4.

## Discussion

### Summary of key findings

Overall, our synthesis of the evidence regarding educational initiatives for professionals involved in self-management support for people with asthma determined that what evidence exists is of unclear or high risk of bias. We synthesised evidence from 15 studies; seven reported at least one positive outcome. Three of six initiatives improved action plan provision/ownership; one of two improved asthma control; and two of seven decreased rates of unscheduled care. All initiatives used educational materials and addressed participants’ knowledge; most used educational meetings and addressed skills. Initiatives with some evidence of effectiveness were more often identified as being explicitly based on clinical practice guidelines; involving local opinion leaders; and including inter-professional education. Effective initiatives were also more likely to have addressed the TDF domains ‘social influences’, ‘environmental context and resources’, ‘behavioural regulation’, ‘beliefs about consequences’, and ‘social/professional role and identity’. It was not possible to draw any conclusions relating to potential links between initiative features and specific outcomes. Due to the complex multi-component nature of these initiatives and the diverse contexts in which they were delivered, it is not possible to conclude with certainty that using these strategies and targeting these domains will result in an intervention being effective. Rather, this initial evidence indicates that these represent plausible approaches and targets for future implementation studies aiming to evaluate the effectiveness of these strategies in improving the provision of supported self-management for asthma.

### Strengths and limitations of this work

The key strengths of this work include the comprehensiveness of the search process. Screening, data extraction, and risk-of-bias assessment were undertaken by two independent reviewers. We used established taxonomies/frameworks to code features of initiatives. However, risk of bias was unclear or high for all studies, although this reflects the wider literature on randomised trials in health professions education.^37^ It is also of note that all included studies took place in high-income countries, which limits the generalisabiity of our findings to other settings. There was often a lack of detail provided regarding the educational initiatives. Additionally, a wide range of outcomes were assessed not only across studies, but within each individual study, with a primary outcome often not specified. The grey literature review was also limited by these problems. This made it challenging to identify common features and classify initiatives as effective or ineffective. We therefore add our voice to a recent call for more detailed and transparent reporting of medical education initiatives,^37^ which can be facilitated by the TIDieR guidelines^38^ and using tools such as the Behaviour Change Technique Taxonomy.^39^

We also reiterate calls for the use of core outcome sets.^40,41^ Inconsistent outcomes precluded meta-analysis, and the lack of implementation outcomes made it difficult to interpret findings. Our retrospective TDF coding enabled us to identify domains that appeared to be targeted by included initiatives, but this may not necessarily reflect the key barriers requiring targeting to facilitate practice change, which context-specific prospective qualitative work would identify.^42,43^ In addition, some EPOC strategies and TDF domains were coded for few or no studies, limiting our ability to asses these. Due to the limited details provided regarding the initiatives, and the fact that initiatives were not developed using the TDF, it was often difficult to identify the TDF domains which appeared to be targeted. The coding of each article was completed independently by two reviewers who then discussed any discrepancies to achieve consensus, involving a third reviewer if necessary. Coding of TDF domains was finalised before interventions were categorised as effective/ineffective.

There is extensive literature on the challenges inherent in synthesising data within systematic reviews of complex interventions.^44–47^ Recent work defines complex interventions are those which have multiple components and causal pathways, and which may also target multiple participants, groups, or organisational levels; require multifaceted adoption, uptake, or integration strategies; or work in a dynamic multidimensional environment.^46^ Therefore, due to the complex nature of the interventions included in our review, care must be taken in interpreting our results and it is not possible to conclude with certainty that using the strategies and targeting the specific domains identified will lead to an effective intervention. Despite these limitations, we identified features common to initiatives with some evidence of effectiveness, which could be further investigated in future implementation studies applying rigorous designs to minimise risk of bias. Indeed, a proposed solution to the challenges in synthesising data on complex interventions is to categorise interventions by key variables and investigate links between these categories and outcomes in the analyses.^44^ The use of theory-based approaches in synthesis of complex interventions is also recommended.^44^ Using theory helps make explicit and testable the associations between interventions and outcomes (i.e. the processes of change), and contributes to building a cumulative science whilst using a common language to synthesise interventions with common features which may be described using disparate language. Here we have used the TDF, a synthesis of decades of research in psychology and behavioural sciences, to identify factors which may form part of these processes of change, which should be explicitly tested in future studies.

### Comparison with existing literature

Asthma care is complex, and there are significant barriers to change at multiple levels. Indeed, the PRISMS meta-review concluded that to implement supported self-management, a whole systems approach is required which addresses patient, professional, and organisational barriers to change.^10^ Professional education is only one of the key elements that contributes to success,^15^ so the inconsistent effectiveness of the included educational initiatives is not unexpected. Indeed, our findings that educational materials and meetings targeting knowledge and skills were features of most initiatives, whether effective or not, indicates that these features may be necessary but not sufficient for professional education to result in clinical behaviour change in this context. Exploring the features of professional education initiatives which have some evidence of effectiveness, as we have done here, is therefore important for informing the design of educational components of future whole-systems implementation strategies.

Educational initiatives including information provision along with additional components were more likely to be effective, largely in accordance with a recent overview of systematic reviews indicating that interventions based on restructuring practice and modifying peer group norms can be effective in changing healthcare professional behaviour.^48^ However, the overview also concluded that interventions also including strategies such as audit and feedback or reminders tended to be more likely to change healthcare professional behaviour.^48^ These aspects were rarely described in the initiatives reviewed here, which may explain their apparent limited effectiveness.

Our findings are in line with the Cochrane review on inter-professional education,^14^ and recent work showing that physicians highly endorse inter-professional management as facilitating PAAP provision.^49,50^ Our review adds to this work by showing that specifically addressing professional roles and responsibilities is a feature more common to successful education for self-management support in asthma. This resonates with our recent qualitative study, which explored the perspectives of professionals on implementing supported self-management for asthma in primary care practices. Demarcation of roles was highlighted as a significant barrier, with nurses frustrated at lack of support from physicians, and physicians recognising this problem but feeling overwhelmed by other priorities.^43^ Suggestions for improvement initiatives included emphasising the evidence for resulting benefits or positive impacts— in line with our finding that effective initiatives tended to target beliefs about consequences—as well as improving teamwork which could be facilitated via team-based education—in line with our finding that effective initiatives tended to include inter-professional education and target social influences and social/professional role and identity.^43^

Previous work has identified multiple barriers to the use of PAAPs in primary care: professionals did not value PAAPs, lacked awareness of when patients could benefit from one, and had difficulty accessing templates.^42^ These barriers may have been overcome in effective initiatives in our review since they involved local respected opinion leaders; were explicitly based on guidelines; demonstrated the positive outcomes for both professionals and patients; and provided template plans for use in practice.

### Recommendations for practice and research

Continuing medical education on self-management support for asthma may benefit from having an inter-professional focus and addressing specific roles and responsibilities which are evolving as new models of care are developing. Many studies measured patient-level health outcomes but not implementation outcomes proximal to the education reflecting change in practice: it is therefore unclear whether a negative outcome is due to an ineffective initiative, or whether additional aspects (such as components aimed at patients, attention to organisational barriers) are required to change patient outcomes. Future research should define and measure a ‘logic pathway’,^51^ as well as intermediate/process outcomes, and explore mechanisms of effect. In addition, advanced statistical methods may be used to examine synergistic and antagonistic effects of initiative components.^52,53^

Alternatively, negative outcomes may be due to initiatives not being delivered as intended.^54^ A range of aspects of fidelity should be investigated in future studies;^54–56^ in particular, assessment of the behaviours targeted by initiatives; using a combination of methods and a mixed-methods approach.^57^ Finally, many strategies and domains were infrequently coded: future research should investigate the impact of barriers to change associated with these domains, and subsequently, the effectiveness of strategies targeting these. For example, the domain ‘memory, attention and decision processes’ was coded only twice, while the EPOC strategy ‘reminders’ was never coded. The only previous review applying the TDF found this to be the most frequently coded domain, suggesting that prompts or aids targeting decision processes were important strategies.^58^ Given that healthcare professional behaviour is likely influenced by underlying automatic/habitual processes as well as reflective ones,^59–61^ it is important to investigate strategies targeting these processes, to inform how guideline-recommended clinical behaviour change for self-management support can best be achieved.

To conclude, in addition to the core components (educational meetings and materials*;* targeting participants’ knowledge and skills), asthma supported self-management education for professionals had a range of additional features. Inter-professional education, and addressing professional roles and identities, were more often coded as present within interventions with some evidence of effectiveness compared to ineffective initiatives. These findings should be interpreted cautiously given that the evidence has unclear or high risk of bias: however, these represent plausible approaches for educational initiatives which should be further investigated as part of whole-systems implementation strategies aiming to improve the provision of supported self-management for asthma.

## Methods

Our methods are summarised here and described in full in our protocol (PROSPERO: 2016:CRD42016032922).^62^ We followed Cochrane recommendations^63^ and PRISMA reporting standards.^64^

This review (as described in our protocol) was run in parallel with a similar review of studies relevant to diabetes. The searches, title/abstract screening, data extraction, and risk of bias assessment were undertaken simultaneously for diabetes and asthma-focussed articles. This paper reports the synthesis of articles focused on asthma-related educational initiatives.

### Eligibility criteria

Eligible studies were RCTs or controlled clinical trials (CCTs) which evaluated educational initiatives on self-management support designed for professionals providing care to people with asthma. We used the Cochrane Effective Practice and Organisation of Care (EPOC) taxonomy of implementation strategies to define ‘educational’ (i.e. either ‘educational materials’; ‘educational meetings’; ‘educational outreach visits or academic detailing’; ‘educational games’; or ‘inter-professional education’).^65^ Studies were included if they could be classified into one of these categories. As a review of an implementation strategy,^51^ primary outcomes included both implementation outcomes reflecting professional behaviour and the process of care (provision/receipt of PAAPs) and health outcomes reflecting disease control (Asthma Control Questionnaire or similar validated measures) and acute events (unscheduled care for asthma). Secondary outcomes were behavioural/cognitive measures related to professionals or patients (e.g. self-efficacy, patient self-care behaviours). Studies were included if they addressed any outcome of interest.

### Information sources and search strategy

Electronic searches were conducted in CENTRAL, MEDLINE, EMBASE, ISI Web of Science, CINAHL, PsycINFO, AMED, Global Health, WHO Global Health Library, ERIC, BNI, RDRB, and Google Scholar for studies published from 1990 (when self-management support for asthma was first recommended in guidelines)^2^ until February 2016 with no language restrictions. A sensitive search strategy was developed following advice from a senior librarian (Marshall Dozier, University of Edinburgh) (see Online Supplementary File 5). Two trial registries were searched (www.clinicaltrials.gov and www.isrctn.com). Reference lists of included studies were screened and authors of included studies were contacted for further information. To update our search prior to publication, forward citation searching for all included studies (a search method shown to be efficient and effective^66^) was completed in May 2017 using Google Scholar.

### Screening

One reviewer (NMc) removed duplicates and clearly irrelevant titles. Three reviewers (NMc, AA, and HP) independently screened a sample of 100 titles and abstracts to clarify the interpretation of inclusion/exclusion criteria. This process was completed twice, until the level of agreement was satisfactory (≥90%). Two reviewers (NMc and AA) then independently screened all titles and abstracts, and the full texts of all potentially eligible studies. Disagreements were resolved by discussion, or arbitration by a third reviewer (HP or ST).

### Data extraction and risk of bias assessment

Two reviewers (NMc and MC; with targeted checks by AA, and arbitration if necessary by HP or ST) independently extracted data onto a piloted form, and assessed risk of bias using the Cochrane Risk of Bias Tool.^67^ Data were extracted on study characteristics, participant characteristics (healthcare professionals and patients), details of the education and control conditions, and relevant outcomes. A summary assessment of the risk of bias (low, high, or unclear) was made for each study using guidance in the Cochrane Handbook.^67^

Headings from the Template for Intervention Description and Replication (TIDieR) checklist^38^ were used to extract general details about the initiatives. We used three taxonomies/frameworks to code specific features of the initiatives as reported in the retrieved articles:

- The EPOC taxonomy to categorise the strategies used to deliver the initiative.^65^
- The Theoretical Domains Framework (TDF), which incorporates 33 theories of behaviour, and 128 corresponding constructs, organised into 14 domains.^68,69^ Although designed to be used prospectively to identify barriers/facilitators to behaviour change which can then be targeted in an implementation strategy, we applied it retrospectively to identify domains that appeared to have been targeted.^58^
- Bloom’s Taxonomy,^70^ to classify the learning objectives or educational goals of the initiatives.

### Data synthesis

Characteristics of the initiatives, and the studies overall, were summarised in descriptive tables. When planning our analyses to evaluate the effectiveness of educational initiatives, we anticipated that heterogeneity between the initiatives assessed and the outcomes evaluated would preclude meta-analysis, and therefore planned a narrative data synthesis. When planning our analyses to determine features of effective initiatives, we anticipated that limited data reporting and heterogeneity would mean that meta-regression or more complex statistical analysis would not be appropriate. We therefore used descriptive data and narrative synthesis to map features to effective/ineffective initiatives.

### Grey literature review

We conducted a review of grey literature, informed by published methodological guidance.^71^ We conducted Google searches, searched the records retrieved in the searches conducted for the systematic review, and searched websites suggested by experts. Screening and data extraction were completed by one reviewer (AB). Risk of bias was assessed using a checklist designed for evaluation of grey literature.^72^ Data were synthesized as described above. Further details on the grey literature review are provided in Online Supplementary File 4.

## Electronic supplementary material

Supplementary Files

## Data Availability

The data that support the findings of this study are available from the corresponding author upon reasonable request.

## Electronic supplementary material

**Supplementary information** accompanies the paper on the *npj Primary Care Respiratory Medicine* website (10.1038/s41533-018-0108-4).
