# Supplementary material for: IMP2ART systematic review of education for healthcare professionals implementing supported self-management for asthma
Source: NPJ Prim Care Respir Med. 2018 Nov 6;28:42. doi: 10.1038/s41533-018-0108-4 (PMC6219611; doi:10.1038/s41533-018-0108-4)
Supplement: Supplementary file 1 — Supplementary Files [file 41533_2018_108_MOESM1_ESM.pdf]

Online supplementary file 1

Table S1: Risk of bias in included studies

Study, year, country	Overall risk of bias rating[a]	Component ratings						
		Random sequence generation	Allocation concealment	Blinding of participants and personnel	Blinding of outcome assessment	Incomplete outcome data	Selective outcome reporting	Other bias
Bruzzese et al 2006 USA	UNCLEAR	UNCLEAR	UNCLEAR	UNCLEAR	LOW	LOW	UNCLEAR	HIGH
Cabana et al 2006 USA	UNCLEAR	LOW	UNCLEAR	LOW	UNCLEAR	UNCLEAR	HIGH	LOW
Clark et al 1998 USA	UNCLEAR	UNCLEAR	UNCLEAR	LOW	UNCLEAR	UNCLEAR	UNCLEAR	LOW
Griffiths et al 2016 UK	UNCLEAR	LOW	LOW	UNCLEAR	LOW	UNCLEAR	LOW	LOW
Shah et al 2011 Australia	UNCLEAR	LOW	LOW	LOW	LOW	LOW	UNCLEAR	UNCLEAR
Cleland et al 2007 UK	UNCLEAR	LOW	UNCLEAR	LOW	LOW	LOW	LOW	HIGH
Cohen et al 2014 Israel	HIGH	HIGH	HIGH	UNCLEAR	LOW	UNCLEAR	LOW	LOW
Evans et al 1997 USA	UNCLEAR	LOW	UNCLEAR	UNCLEAR	UNCLEAR	UNCLEAR	UNCLEAR	LOW
Homer et al 2005 USA	HIGH	HIGH	UNCLEAR	UNCLEAR	LOW	LOW	UNCLEAR	UNCLEAR
Prabhakaran et al 2012 Singapore	UNCLEAR	UNCLEAR	UNCLEAR	UNCLEAR	UNCLEAR	LOW	LOW	LOW
Sheikh et al 2016 USA	UNCLEAR	UNCLEAR	UNCLEAR	UNCLEAR	LOW	LOW	LOW	LOW
Smeele et al 1999 Netherlands	UNCLEAR	UNCLEAR	UNCLEAR	UNCLEAR	UNCLEAR	LOW	UNCLEAR	LOW
Toelle et al 1993 Australia	HIGH	HIGH	HIGH	UNCLEAR	UNCLEAR	LOW	UNCLEAR	LOW
Tomson et al 1997 Sweden	HIGH	HIGH	HIGH	UNCLEAR	UNCLEAR	HIGH	UNCLEAR	LOW
Volovitz et al 2003 Israel	HIGH	HIGH	HIGH	UNCLEAR	UNCLEAR	UNCLEAR	HIGH	LOW

Note: [a]Assessment of overall risk of bias (low, high, or unclear) was made for each study using guidance in the Cochrane Handbook (low = low risk of bias rating for all key components; high = high risk of bias rating for one or more key components; unclear = unclear risk of bias rating for one or more key components)

Risk of bias assessment notes for includes studies

Source: Higgins JPT, Green S (editors). Cochrane Handbook for Systematic Reviews of Interventions. Version 5.1.0 [updated March 2011]. The Cochrane Collaboration, 2011. Available at: www.cochrane-handbook.org

Bruzzese et al 2006 USA:

Domain	Risk of bias				Support for judgment
	Low	High	Unclear	Not applicable	
Random sequence generation *(selection bias)*	☐	☐	☒	☐	Sequence generation process nr
Allocation concealment *(selection bias)*	☐	☐	☒	☐	nr
Blinding of participants and personnel *(performance bias)*	☐	☐	☒	☐	nr
Blinding of outcome assessment *(detection bias)*	☒	☐	☐	☐	NERI, an independent research company blind to treatment status, conducted caregiver telephone interviews at baseline and at 12- and 24-months postbaseline.
Incomplete outcome data *(attrition bias)*	☒	☐	☐	☐	Baseline: 307 I; 284 C<br>12 months: 244 I (79%); 228 C (80%)<br>24 months: 195 I (64%); 173 C (61%)<br>Similar attrition across groups
Selective outcome reporting *(reporting bias)*	☐	☐	☒	☐	Unclear reporting of p-values (within-group vs. between-group differences) also specific outcomes not clearly outlined in methods
Other bias	☐	☒	☐	☐	While the school nurse provided asthma education as needed, a study limitation is that students in the treatment and control groups received OAS. During the first year of the study, OAS was offered to all third and fifth grade students with asthma in NYC public schools, which may have confounded our results. Improvements experienced by control students may have been due, in part, to their participation in OAS, thereby minimizing the benefits of

					our intervention.
Overall risk of bias	☐	☐	☒	☐	
Notes:					

Cabana et al 2006 USA:

**Domain**	**Risk of bias**				**Support for judgment**
	**Low**	**High**	**Unclear**	**Not applicable**	
Random sequence generation *(selection bias)*	☒	☐	☐	☐	Because physicians who are exposed to the intervention might disseminate new information to other physicians, we randomized by site versus randomizing by physician to prevent the possibility of contamination. We matched each of the 10 sites into 5 similar pairs on the basis of population, asthma prevalence, percentage of the population that is Hispanic and/or black, climate, and managed care penetration in the health care market. Within each pair, using a coin toss, we randomly selected 1 site as a control and 1 site for the intervention.
Allocation concealment *(selection bias)*	☐	☐	☒	☐	nr
Blinding of participants and personnel *(performance bias)*	☒	☐	☐	☐	Patients and their parents were blind to physicians' involvement in the intervention. Physicians were blinded to which patients were selected for the survey.
Blinding of outcome assessment *(detection bias)*	☐	☐	☒	☐	Nr Not enough detail provided: unclear if interviewers blinded, GPs reporting on own behaviour
Incomplete outcome data *(attrition bias)*	☐	☐	☒	☐	Adequate follow-up rates, similar across groups, but numbers contributing to analyses not clear
Selective outcome reporting *(reporting bias)*	☐	☒	☐	☐	Outcomes assessed via multiple items: not all listed: only selected ones reported: potential for bias?
Other bias	☒	☐	☐	☐	
Overall risk of bias	☐	☐	☒	☐	
Notes:					

Clark et al 1998 USA:

**Domain**	**Risk of bias**				**Support for judgment**
	**Low**	**High**	**Unclear**	**Not applicable**	
Random sequence generation *(selection bias)*	☐	☐	☒	☐	Physicians 'randomly assigned to program or control status' (p.832); no further details provided
Allocation concealment *(selection bias)*	☐	☐	☒	☐	No details provided
Blinding of participants and personnel *(performance bias)*	☒	☐	☐	☐	"Patients and their parents were blind to physicians' involvement in the intervention." (p.832)
Blinding of outcome assessment *(detection bias)*	☐	☐	☒	☐	No details provided (important in this context since all outcomes self/parent-reported to outcome assessors)<br><br>Physicians received no information concerning the questions to be addressed to their patients.<br><br>NOT ENOUGH DATA TO DECIDE
Incomplete outcome data *(attrition bias)*	☐	☐	☒	☐	Missing data not addressed
Selective outcome reporting *(reporting bias)*	☐	☐	☒	☐	No study protocol; full list of specific outcomes not provided; outcomes and their measurement unclear
Other bias	☒	☐	☐	☐	
Overall risk of bias	☐	☐	☒	☐	
Notes:					

Griffiths et al 2016 UK:

**Domain**	**Risk of bias**				**Support for judgment**
	**Low**	**High**	**Unclear**	**Not applicable**	
Random sequence generation *(selection bias)*	☒	☐	☐	☐	
Allocation concealment *(selection bias)*	☒	☐	☐	☐	
Blinding of participants and personnel *(performance bias)*	☐	☐	☒	☐	
Blinding of outcome assessment *(detection bias)*	☒	☐	☐	☐	

Incomplete outcome data *(attrition bias)*	☐	☐	☒	☐	
Selective outcome reporting *(reporting bias)*	☒	☐	☐	☐	
Other bias	☒	☐	☐	☐	
Overall risk of bias	☐	☐	☒	☐	
Notes:					

Shah et al 2011 Australia:

**Domain**	**Risk of bias**				**Support for judgment**
	**Low**	**High**	**Unclear**	**Not applicable**	
Random sequence generation *(selection bias)*	☒	☐	☐	☐	Randomly allocated to intervention or control group by minimisation within the strata of sex and Fellowship of the Royal Australian College of General Practitioners using a computer-generated algorithm<br>If >1 GP recruited at a practice, first to be recruited was randomly allocated, others in the practice allocated to the same group
Allocation concealment *(selection bias)*	☒	☐	☐	☐	Concealment<br>of randomisation was maintained<br>until GP characteristics were entered into a database and a randomisation<br>code was generated.
Blinding of participants and personnel *(performance bias)*	☒	☐	☐	☐	The GPs enrolled in the study, the<br>parents and carers of patients enrolled<br>in the study, and the project officer<br>administering the parent questionnaire<br>interviews were blinded to group allocation.<br>GPs were informed that the study would involve completion of two questionnaires and participation in two workshops within 3 years. GPs in the<br>control group were offered the PACE<br>workshops at the end of the study<br>period.
Blinding of outcome assessment *(detection bias)*	☒	☐	☐	☐	
Incomplete outcome data *(attrition bias)*	☒	☐	☐	☐	Follow-up rates acceptable and similar across groups

					Numbers contributing to each analysis clearly outlined
Selective outcome reporting *(reporting bias)*	☐	☐	☒	☐	
Other bias	☐	☐	☒	☐	Study underpowered
Overall risk of bias	☐	☐	☒	☐	
Notes:					

Cleland et al 2007 UK:

**Domain**	**Risk of bias**				**Support for judgment**
	**Low**	**High**	**Unclear**	**Not applicable**	
Random sequence generation *(selection bias)*	☒	☐	☐	☐	p312 "assigned by an independent statistician, blind to the identity of the practices, to intervention (n=6) or control (n=7), using random number tables."
Allocation concealment *(selection bias)*	☐	☐	☒	☐	method of concealment is not described
Blinding of participants and personnel *(performance bias)*	☒	☐	☐	☐	Blinding not reported, but unlikely to influence outcome as outcomes were all at patient level while intervention at nurse level
Blinding of outcome assessment *(detection bias)*	☒	☐	☐	☐	Blinding not reported, but unlikely to influence outcome as 2x outcomes were objective measures of asthma medication, and other 2 were self-reported by patients not in presence of outcome assessors
Incomplete outcome data *(attrition bias)*	☒	☐	☐	☐	Missing outcome data balanced in numbers across intervention groups, with similar reasons for missing data across groups:<br><br>p315 "78/106 (74%) were returned by control practice patients and 99/130 (76%) from intervention practice patients."
Selective outcome reporting *(reporting bias)*	☒	☐	☐	☐	outcomes clearly described and all results reported
Other bias	☐	☒	☐	☐	STUDY RESULTS SIGNIFICANT FOR PRIMARY OUTCOME, BUT WAS UNDERPOWERED TO DETECT THIS

					Potential source of selection bias: differences between those who did & did not return questionnaire: those who did return, tended to be older, female, more affluent, lower medication use
Overall risk of bias	☐	☐	☒	☐	
Notes:					

Cohen et al 2014 Israel:

**Domain**	**Risk of bias**				**Support for judgment**
	**Low**	**High**	**Unclear**	**Not applicable**	
Random sequence generation *(selection bias)*	☐	☒	☐	☐	No randomisation
Allocation concealment *(selection bias)*	☐	☒	☐	☐	No allocation concealment
Blinding of participants and personnel *(performance bias)*	☐	☐	☒	☐	nr
Blinding of outcome assessment *(detection bias)*	☒	☐	☐	☐	Nr<br>Outcome data at level of patient rather than physician, routine data analysis
Incomplete outcome data *(attrition bias)*	☐	☐	☒	☐	Attrition and missing data unclear
Selective outcome reporting *(reporting bias)*	☒	☐	☐	☐	One outcome, clearly outlined
Other bias	☒	☐	☐	☐	
Overall risk of bias	☐	☒	☐	☐	
Notes:					

Evans et al 1997 USA:

**Domain**	**Risk of bias**				**Support for judgment**
	**Low**	**High**	**Unclear**	**Not applicable**	
Random sequence generation *(selection bias)*	☒	☐	☐	☐	To convince BCH staff that the choice of which clinics received<br>the intervention was not influenced by any other factors, we<br>randomly allocated one panel to intervention status by asking a<br>volunteer to toss a coin during a meeting of BCH supervisors and administrators.
Allocation concealment *(selection bias)*	☐	☐	☒	☐	

Blinding of participants and personnel *(performance bias)*	☐	☐	☒	☐	nr
Blinding of outcome assessment *(detection bias)*	☐	☐	☒	☐	nr
Incomplete outcome data *(attrition bias)*	☐	☐	☒	☐	Exclusions/drop-outs unclear
Selective outcome reporting *(reporting bias)*	☐	☐	☒	☐	
Other bias	☒	☐	☐	☐	
Overall risk of bias	☐	☐	☒	☐	
Notes:					

Homer et al 2005 USA:

**Domain**	**Risk of bias**				**Support for judgment**
	**Low**	**High**	**Unclear**	**Not applicable**	
Random sequence generation *(selection bias)*	☐	☒	☐	☐	No details on sequence generation provided<br><br>Contamination risk (see below)
Allocation concealment *(selection bias)*	☐	☐	☒	☐	No details on allocation concealment provided
Blinding of participants and personnel *(performance bias)*	☐	☐	☒	☐	P465 Participating practices and the faculty who coached them were aware of their group assignment<br><br>Blinding not possible, however, not clear the extent to which this could have influenced outcomes (assessed at patient level)
Blinding of outcome assessment *(detection bias)*	☒	☐	☐	☐	P465 Parents and their interviewers were unaware of randomization status.
Incomplete outcome data *(attrition bias)*	☒	☐	☐	☐	High follow-up rates in both groups
Selective outcome reporting *(reporting bias)*	☐	☐	☒	☐	
Other bias	☐	☐	☒	☐	P467 The risk of some degree of contamination between intervention and control practices was real<br><br>Problems with intervention adherence
Overall risk of bias	☐	☒	☐	☐	Contamination risk
Notes:					

Prabhakaran et al 2012 Singapore:

**Domain**	**Risk of bias**				**Support for judgment**
	**Low**	**High**	**Unclear**	**Not applicable**	
Random sequence generation *(selection bias)*	☐	☐	☒	☐	Not described
Allocation concealment *(selection bias)*	☐	☐	☒	☐	Not described
Blinding of participants and personnel *(performance bias)*	☐	☐	☒	☐	Not described
Blinding of outcome assessment *(detection bias)*	☐	☐	☒	☐	Not described
Incomplete outcome data *(attrition bias)*	☒	☐	☐	☐	No missing data; similar high follow-up rates across groups
Selective outcome reporting *(reporting bias)*	☒	☐	☐	☐	
Other bias	☒	☐	☐	☐	
Overall risk of bias	☐	☐	☒	☐	
Notes:					

Sheikh et al 2016 USA:

**Domain**	**Risk of bias**				**Support for judgment**
	**Low**	**High**	**Unclear**	**Not applicable**	
Random sequence generation *(selection bias)*	☐	☐	☒	☐	
Allocation concealment *(selection bias)*	☐	☐	☒	☐	
Blinding of participants and personnel *(performance bias)*	☐	☐	☒	☐	
Blinding of outcome assessment *(detection bias)*	☒	☐	☐	☐	
Incomplete outcome data *(attrition bias)*	☒	☐	☐	☐	
Selective outcome reporting *(reporting bias)*	☒	☐	☐	☐	
Other bias	☒	☐	☐	☐	
Overall risk of bias	☐	☐	☒	☐	
Notes:					

Smeele et al 1999 Netherlands:

Domain	Risk of bias				Support for judgment
	Low	High	Unclear	Not applicable	
Random sequence generation *(selection bias)*	☐	☐	☒	☐	GPs in the same local group were preferably allocated to the same education group. These groups were allocated at random to the experimental and control modality of the study.
Allocation concealment *(selection bias)*	☐	☐	☒	☐	nr
Blinding of participants and personnel *(performance bias)*	☐	☐	☒	☐	Nr
Blinding of outcome assessment *(detection bias)*	☐	☐	☒	☐	Nr
Incomplete outcome data *(attrition bias)*	☒	☐	☐	☐	Drop-out acceptable (although unable to compare groups), numbers in each analysis clearly outlined
Selective outcome reporting *(reporting bias)*	☐	☐	☒	☐	
Other bias	☒	☐	☐	☐	
Overall risk of bias	☐	☐	☒	☐	
Notes:					

Toelle et al 1993 Australia:

Domain	Risk of bias				Support for judgment
	Low	High	Unclear	Not applicable	
Random sequence generation *(selection bias)*	☐	☒	☐	☐	No random allocation
Allocation concealment *(selection bias)*	☐	☒	☐	☐	No allocation concealment
Blinding of participants and personnel *(performance bias)*	☐	☐	☒	☐	Nr
Blinding of outcome assessment *(detection bias)*	☐	☐	☒	☐	Nr
Incomplete outcome data *(attrition bias)*	☒	☐	☐	☐	High follow-up rates across both groups, numbers contributing to each analysis clearly stated
Selective outcome reporting *(reporting bias)*	☐	☐	☒	☐	

Other bias	☒	☐	☐	☐	
Overall risk of bias	☐	☒	☐	☐	
Notes:					

Tomson et al 1997 Sweden:

**Domain**	**Risk of bias**				**Support for judgment**
	**Low**	**High**	**Unclear**	**Not applicable**	
Random sequence generation *(selection bias)*	☐	☒	☐	☐	No randomisation
Allocation concealment *(selection bias)*	☐	☒	☐	☐	No allocation concealment
Blinding of participants and personnel *(performance bias)*	☐	☐	☒	☐	nr
Blinding of outcome assessment *(detection bias)*	☐	☐	☒	☐	nr
Incomplete outcome data *(attrition bias)*	☐	☒	☐	☐	Fewer responses in control group
Selective outcome reporting *(reporting bias)*	☐	☐	☒	☐	
Other bias	☒	☐	☐	☐	
Overall risk of bias	☐	☒	☐	☐	
Notes:					

Volovitz et al 2003 Israel:

**Domain**	**Risk of bias**				**Support for judgment**
	**Low**	**High**	**Unclear**	**Not applicable**	
Random sequence generation *(selection bias)*	☐	☒	☐	☐	No randomisation
Allocation concealment *(selection bias)*	☐	☒	☐	☐	No randomisation
Blinding of participants and personnel *(performance bias)*	☐	☐	☒	☐	Blinding not addressed
Blinding of outcome assessment *(detection bias)*	☐	☐	☒	☐	Blinding not addressed
Incomplete outcome data *(attrition bias)*	☐	☐	☒	☐	Attrition/exclusions/missing data not addressed
Selective outcome reporting *(reporting bias)*	☐	☒	☐	☐	Np protocol referred to, no primary outcome specified, not clear that report includes all expected outcomes Results poorly and incompletely reported

Other bias	☒	☐	☐	☐	
Overall risk of bias	☐	☒	☐	☐	
Notes:					

Online supplementary file 2

Table S2: Characteristics of included studies

Study, year, country	Design, risk of bias	Setting, duration	Participants analysed/ recruited	Brief intervention description	Control condition	Key outcomes assessed
STUDIES WHICH EVALUATED THE PACE PROGRAMME						
Bruzzese et al 2006<br>USA	2-group cluster RCT<br><br>Unclear	44 schools = clusters<br><br>Target: low-income ethnic minority families<br><br>2yr	PCPs: NR<br><br>Children/families: 368 (195 I, 173 C) 2yr/ 472 1yr (244 I, 228 C)/ 591 (307 I, 284 C)	PACE educational sessions on: preventive therapy, communication and patient education strategies, procedures for establishing medication plans in schools;<br>Guideline-based sample treatment plans based on asthma severity, blank treatment plans;<br>Encouragement to complete required forms when medication needed at school;<br>CME credits and catered meals;<br>Invitation letter signed by Commissioner of Health	Standard care control	Primary outcome not specified;<br>Urgent physician visits;<br>ED visits;<br>Hospitalizations
Cabana et al 2006<br>USA	2-group cluster RCT<br><br>Unclear	10 cities/ regions = clusters<br><br>Primary care practice<br><br>2yr	Paediatricians: 66 2yr/ 76 1yr/ 94 (51 I, 43 C)<br><br>Children/parents: 693 (439 I, 254 C) 2yr/ 731 (363 I; 368 C) 1yr/ 870 (418 I; 452 C)	PACE program: interactive seminars to review asthma guidelines, specific communication techniques, and key asthma educational messages;<br>Protocol for patient communication self-assessment, sample action plans, materials to use with patients;<br>CME credits, certificate, honorarium	Standard care control (received honorarium)	Primary outcome not specified;<br>Physician self-efficacy for reviewing long-term care plan with parents;<br>Physician self-efficacy for developing a short-term plan for asthma;<br>Caregiver's Perception of Doctor's Care;<br>Parent was asked if child met specific goals;<br>Change in number of urgent office visits;<br>Change in number of ED visits;<br>Change in number of hospitalizations
Clark et al 1998<br>USA	2-group RCT<br><br>Unclear	74 practices<br><br>2yr	Paediatricians: 67 (34 I, 33 C) 2yr/ 69 22 months/ 74 (38 I, 36 C)<br><br>Children/parents: 369 (202 I, 167 C) 2yr/ 472 22 months/ 637 (336 I, 301 C)	PACE program: interactive seminars to develop skills in treating childhood asthma; educating families on self-management; developing a partnership with patients;<br>Protocol for patient communication self-assessment, sample action plan, materials to use with patients	Standard care control	Primary outcome not specified;<br>Parent report: receipt of written plan to adjust dose when symptoms change;<br>Physician report: write down how to adjust medicine when symptoms change;<br>Physician report: provide guidelines to adjust therapy when conditions change;<br>ED visits;<br>Hospitalizations;<br>Follow-up office visits after episode of symptoms

Study, year, country	Design, risk of bias	Setting, duration	Participants analysed/ recruited	Brief intervention description	Control condition	Key outcomes assessed
STUDIES WHICH EVALUATED THE PACE PROGRAMME						
Griffiths et al 2016<br>UK	2-group cluster RCT<br><br>Unclear	84 primary care practices = clusters<br><br>1yr	Nurses & GPs: NR<br><br>South Asian adults and children: 336 (168 I, 168 C)/ 375 (183 I, 192 C)	Adapted PACE program for UK clinicians caring for south Asian patients: two seminars (lunchtime office-based visits) using a DVD, led by asthma experts; summaries of clinical guidelines; promoted key messages for successful asthma care; video-recorded examples of consultations illustrating culturally-specific issues about asthma and communication problems, and modelled potential solutions<br>Copy of DVD and manual provided<br>Participants encouraged to practice skills learned at the first session, which were reviewed at the second	Standard care control	PRIMARY OUTCOME: Proportion of patients without unscheduled care;<br>PRIMARY OUTCOME: Time to first unscheduled contact with an asthma exacerbation;<br><br>Asthma-related QoL (AQ20);<br>Asthma-related self-efficacy
Shah et al 2011<br>Australia	2-group RCT<br><br>Unclear	109 primary care practices<br><br>1yr	GPs: 106 (57 I, 49 C)/ 150 (78 I, 72 C)<br><br>Children/parents: 213 (106 I; 107 C)/ 221 (111 I, 110 C)	Adapted PACE program: interactive workshops on Asthma Cycle of Care, assessment of pattern of asthma, appropriate use of medications, provision of action plan, communication and education strategies	Standard care control	PRIMARY OUTCOME: Parent report: written asthma action plan received 1+ times<br><br>GP confidence in using 10 communication strategies;<br>GP report: written asthma action plan provided >70% of the time;<br>1+ hospital visits;<br>ICS use;<br>LABA use
STUDIES WHICH EVALUATED INITIATIVES OTHER THAN PACE						
Cleland et al 2007<br>UK	2-group cluster RCT<br><br>Unclear	13 primary care practices = clusters (6 I, 7 C)<br><br>6 months	Nurses: NR<br><br>Adult patients: routine data analyses: 629 (366 I, 263 C)/ 629 (373 I; 256 C) questionnaire: 177 (99 I, 78 C)/ 236 (130 I, 106 C)	One interactive seminar on clinical and communication skills for asthma management;<br>Focus on use of asthma action plans within consultation;<br>Examples of effective communication strategies;<br>Patient resources	Standard care control	PRIMARY OUTCOME: Asthma-related QoL (Mini AQLQ)<br><br>Asthma control (ACQ);<br>Number of oral steroid courses;<br>Number of β2-agonist inhaler prescriptions

Study, year, country	Design, risk of bias	Setting, duration	Participants analysed/ recruited	Brief intervention description	Control condition	Key outcomes assessed
STUDIES WHICH EVALUATED THE PACE PROGRAMME						
Cohen et al 2014 Israel	5-group CCT<br><br>High	Divisions of health maintenance organization = 5 'clusters'<br><br>Primary care practice<br><br>2yr	GPs: analysed NR/ 258 (45 I1, 35 I2, 21 I3, 36 I4, 121 C)<br>Nurses: NR<br><br>Patients >12yr with uncontrolled asthma in area: 935 (46 I1, 176 I2, 79 I3, 115 I4, 519 C) 2yr/ 971 (49 I1, 193 I2, 85 I3, 125 I4, 519 C) 1yr/ 1056 (54 I1, 219 I2, 106 I3, 171 I4, 506 C)	I1: GPs: Received: list of patients with uncontrolled asthma, request to meet with patients to consider ways of improving treatment, lecture on asthma treatment innovations<br>I2:GPs: I1 plus workshop: lectures on principles of educating patients for self-monitoring, symptom control, healthy lifestyle, environmental changes; training in use of different kinds of inhalers<br>I3: GPs & nurses: I1 plus simulation training: practice clinical and communication skills with simulated patients<br>Topics: taking medical histories from patients who do not easily express complaints; identifying and addressing reasons patients lose control, promoting adherence to therapy, regular medical appointments, healthy lifestyle<br>I4: GPs & nurses: I1 plus I2 plus I3	Standard care control	PRIMARY (AND ONLY) OUTCOME: Change in rate of uncontrolled asthma (based on inhaler purchase data)
Evans et al 1997 USA	2-group cluster RCT<br><br>Unclear	Clusters = 2 panels of 11 clinics<br>22 primary care practices (11 I, 11 C)<br><br>2yr	Practice team: analysed NR/ 134 (80 I, 54 C)<br><br>Children/caregivers: 245 (176 I, 69 C)/ 358	Creating a Medical Home for Asthma;<br>Learning sessions to introduce program, outline roles of team members, emphasize roles of support staff, review communication skills and family education, introduce asthma screening process;<br>Guideline-based treatment protocols; medical record forms; CME credit<br>Tutorial session observing physicians treating children with asthma; monthly visits to practices by a full-time nurse educator to help solve problems; telephone consultation for difficult problems	Standard care control (but received guidelines, and supplies of same medications and delivery devices)	Primary outcome not specified;<br>Caregiver received education by physician/nurse on written treatment plan;<br>Caregiver received education by physician/nurse (Patient Education Index);<br>Received any β-agonist;<br>Received any inhaled anti-inflammatory;<br>Received oral steroid
Homer et al 2005 USA	2-group cluster RCT<br><br>High	43 practices = clusters (22 I, 21 C)<br><br>1yr	3-member practice team (physician, nurse, front office staff): NR<br><br>Children/parents: 490 (236 I, 254 C)/ 631 (294 I, 337 C)	Learning collaborative project<br>Practices identified performance gaps in their practice;<br>Learning sessions on Chronic Care Model, Model for Improvement (quality improvement approach);<br>Guidelines with support tools (encounter forms and an electronic patient registry);<br>Support through additional learning sessions, conference calls, e-mail list, expert review and performance feedback	Standard care control	PRIMARY OUTCOME: Written asthma management plan received in past 12 months;<br>PRIMARY OUTCOME: Daily use of inhaled steroid medication in past 4 weeks;<br>PRIMARY OUTCOME: Daily use of controller medication in past 4 weeks;<br>Hospitalization in past 12 months;<br>ED visit in past 12 months;<br>Asthma attack in past 12 months

Study, year, country	Design, risk of bias	Setting, duration	Participants analysed/ recruited	Brief intervention description	Control condition	Key outcomes assessed
STUDIES WHICH EVALUATED THE PACE PROGRAMME						
Prabhakaran et al<br>2012<br>Singapore	3-group RCT<br><br>Unclear	1 tertiary hospital<br><br>3 months	Enrolled nurses: 145 (47 I1, 51 I2, 47 I3)/ 162 (59 I1, 55 I2, 48 I3)<br><br>No patients recruited	I1: Workshop on asthma management, followed by demonstration, hands-on practice, assessment on various inhaler techniques<br>I2: As I1 except lectures in e-learning format<br>I3: combination of I1 and I2	No control group; intervention groups compared	Primary outcome not specified;<br>Knowledge
Sheikh et al<br>2016<br>USA	2-group cluster RCT<br><br>Unclear	10 primary care paediatric practices = clusters (5 I, 5 C)<br><br>2yr	GPs, nurses practitioners, nurses, medical assistants: NR<br><br>Patients: NR (outcomes assessed via medical record review, focus on overall practice evaluation)	Training of Asthma Leaders in each practice<br>Curriculum covered guidelines, asthma action plans, severity-specific diagnosis of asthma, medication teaching, anti-inflammatory medication use, follow-up and appropriate sub-specialty consultation practices: 1-hr sessions/week for 6 weeks, plus 1-day course for American Lung Association Asthma Caregiver Certification<br>Monthly in-office, one-on-one tutoring sessions in each practice between Asthma Leaders and a registered respiratory therapist, with physician attendance requested<br>Therapist also available for telephone consultations<br>Monthly meetings between therapist and paediatric pulmonologist to review updates of the meetings	Control group received intervention for 6 months, after the initial 1yr follow-up	Primary outcome not specified;<br>Proportion of medical records with documentation of quality asthma care indicator:<br>Asthma education;<br>Asthma action plan;<br>Acute care visit
Smeele et al<br>1999<br>Netherlands	2-group cluster RCT<br><br>Unclear	GPs in same 'local group' = cluster<br>Primary care practice<br><br>1yr	GPs: 34 (17 I, 17 C)/ 34<br>Practice assistants: NR<br><br>Adult asthma or COPD patients: 433 (210 ,I 223 C)/ 544	**FOCUS ON ASTHMA AND COPD**<br>Group education and peer review program focused on gaps in adherence to guidelines identified in findings of the baseline measurement, various strategies for implementing guidelines used, skills training and peer review of performance;<br>Educational session for practice assistants focusing on knowledge of asthma/COPD, peak flow measurement, and inhalation instructions	Standard care control	Primary outcome not specified; includes asthma and COPD;<br>Knowledge score (0-10) on diagnosis and treatment of acute severe asthma and exacerbations;<br>Patient report received written patient education;<br>No. patients reporting symptom exacerbations past 3 months (ratio)
Toelle et al<br>1993<br>Australia	2-group CCT<br><br>High	Two areas = 2 'clusters'<br>Primary schools<br><br>9 months	GPs of recruited children, local pharmacists, community nurses: NR	Evening workshops on management principles based on a consensus plan (GPs and pharmacists); suggested management plans for patients provided (GPs); importance of advising about appropriate management emphasized (pharmacists); in-service education session at work place (nurses)<br>No-intervention control	Standard care control	Primary outcome not specified;<br>Unscheduled doctor/ ED visits;<br>Symptoms limiting activity;<br>Parent knowledge of asthma score

Study, year, country	Design, risk of bias	Setting, duration	Participants analysed/ recruited	Brief intervention description	Control condition	Key outcomes assessed
STUDIES WHICH EVALUATED THE PACE PROGRAMME						
			Children/families: 120 (65 I, 55 C)/ 132 (72 I, 60 C)			
Tomson et al 1997 Sweden	2-group CCT<br><br>High	2 areas of Stockholm County = 'clusters'<br>30 primary care practices (21 I, 9 C)<br><br>18 months	GPs: 63 (44 I, 19 C)/ NR<br><br>Patients (whether child/ adult unclear): 157 (138 I, 19 C)/ 331 (249 I, 82 C)	Academic detailing for diagnosis and treatment of asthma:<br>Generated relevant topics by direct contact with GPs; information package based on guidelines, handbooks and literature; presented in a monthly/bimonthly local leaflet and a 45-min oral presentation at practices; two visits per year to practices, GPs discussed treatment of asthma	Standard care control	Primary outcome not specified;<br>Advice to use PEF meter in a self-management plan provided;<br>Amount of inhaled steroids taken increased;<br>Ratios of prescribed inhaled β-agonists to inhaled glucocorticoids
Volovitz et al 2003 Israel	4-group CCT<br><br>High	Two regions in central Israel Health Maintenance Organization Primary care practice<br><br>9 months	GPs & paediatricians: NR<br><br>Adult & child patients: 4233 (132 I1, 788 I2, 1388 C1, 1925 C2)/ NR	I1: Asthma education program on the pathophysiology, clinical and psychosocial aspects of asthma, asthma management and prevention; emphasis on identifying potential obstacles to patient adherence and ways of building a good patient-physician relationship; guidelines distributed; application of learning to future consultations (follow-up of patients according to protocol) monitored<br>I2: I1 except follow-up not monitored	C1: No intervention, patients in same region<br>C2: No intervention, patients in different region	Primary outcome not specified;<br>Acute asthma intervention plan ownership;<br>ICS/ LABA/ Leukotriene antagonist (Singulair)/ SABA use;<br>Improvement in shortness of breath symptoms;

Note: ACQ = asthma control questionnaire; AQ20 = Airways Questionnaire 20; AQLQ = Asthma Quality of Life Questionnaire; CCT = controlled clinical trial; C = control group; CME = continuing medical education; COPD = chronic obstructive pulmonary disease; ED = emergency department; GP = general practitioner; I = intervention group; ICS = inhaled corticosteroid; LABA = long-acting β-agonist; NR = not reported; PACE = Physician Asthma Care Education; PCP = primary care physician; PEF = peak expiratory flow; QoL = quality of life; RCT = randomized controlled trial; SABA = short-acting β-agonist

Online supplementary file 3

Table S3: Effectiveness of included educational initiatives

Study, year, country	Design, risk of bias	Intervention summary	Key results	Conclusions
Bruzzese et al 2006<br>USA	2-group cluster RCT<br><br>Unclear	PACE initiative: develops skills for treating asthma, including supporting patients & families to self-manage<br><br>Standard care control	Primary outcome not specified; all participants followed up included in all analyses<br>Implementation outcomes NR<br>Parent-report, Mean (SD)<br>**Urgent physician visits** 1yr: I: 2.1 (3.5), C: 3.2 (13.8); 2yr: I: 1.7 (3.0), C: 1.8 (3.6) p=NS for both<br>**ED visits** 1yr: I: 1.0 (1.8), C: 1.3 (3.2) 2yr: I: 0.9 (2.2), C: 0.9 (1.8) p=NS for both<br>**Hospitalizations** 1yr: I: 0.1 (0.4). C: 0.1 (0.6) p=NS 2yr: I: 0.2 (0.6), C: 0.1 (0.3) p<.05 (favours C)	No evidence of effectiveness
Cabana et al 2006<br>USA	2-group cluster RCT<br><br>Unclear	PACE initiative: develops skills for treating asthma, including supporting patients & families to self-manage<br><br>Standard care control (received honorarium)	Primary outcome not specified; numbers in each analysis NR<br>Implementation outcomes<br>Multiple items used to assess self-efficacy and outcome expectancy: data NR<br>**Physician self-efficacy for reviewing long-term care plan with parents**<br>1yr: I: OR 3.72 (95% CI 1.17 to 11.7), C: REF, p=NR (favours I)<br>**Physician self-efficacy for developing a short-term plan for asthma**<br>1yr: I: OR: 3.79 (95% CI 1.34 to 10.8), C: REF p=NR (favours I)<br><br>Multiple items used: data NR; Items at 2yr not same as items at 1yr<br>**Caregiver's Perception of Doctor's Care**<br>Overall index: 2yr: I: OR 0.10 (95% CI 0.01 to 0.20), C: REF, p=.023 (favours I)<br>**Parent was asked if child met specific goals**<br>1yr: I: OR 1.50 (95% CI 1.02 to 2.24), C: REF, p=NR (favours I)<br>Parent-report, Mean<br>**Change in number of urgent office visits per yr** 1yr: I: -1.07, C: -0.9, p=NS; 2yr: p=.602<br>**Change in number of ED visits per yr** 1yr: I: -0.55, C: -0.30, p<.05; 2yr: p=.038<br>**Change in number of hospitalizations per yr** 1yr: I: -0.06, C: -0.06, p=NS; 2yr: p=.833	Some evidence of effectiveness
Clark et al 1998<br>USA	2-group RCT<br><br>Unclear	PACE initiative: develops skills for treating asthma, including supporting patients & families to self-manage<br><br>Standard care control	Primary outcome not specified; numbers in each analysis NR<br>Selected outcomes assessed at 5/22 months also reported at 2yr<br>Implementation outcomes<br>**Parent report: receipt of written plan to adjust dose when symptoms change**<br>22 months: I: 26%, C: 16%, OR 1.74, p=.03<br>**Physician report: write down how to adjust medicine when symptoms change**<br>5 months: scale mean: I: 4.30, C: 3.46, p=.001; 2yr p=.05<br>**Physician report: provide guidelines to adjust therapy when conditions change**<br>5 months: I: 80.39%, C: 65.34%, p=.003; 2yr: p=.06	Some evidence of effectiveness

Study, year, country	Design, risk of bias	Intervention summary	Key results	Conclusions
			Parent-report, Mean<br>**ED visits** 22 months: I: 0.65, C: 0.67, p=NS; 24 months: I: 0.29, C: 0.47, p=.44<br>**Hospitalizations** 22 months I: 0.08, C: 0.08, p=NS; 24 months: I 0.03, C 0.10, p=.03<br>**Follow-up office visits after episode of symptoms**<br>22 months: I: 0.94, C: 1.61, p=.005; 24 months: I 0.59, C 0.71, p=.36	
Griffiths et al<br>2016<br>UK	2-group cluster RCT<br><br>Unclear	PACE initiative: develops skills for treating asthma, including supporting patients & families to self-manage<br><br>Standard care control	Implementation outcomes NR<br>**PRIMARY OUTCOMES**<br>Medical record review, N=358<br>**Proportion of patients without unscheduled care** OR 0.71 (95% CI 0.43 to 1.20), p=0.202<br>**Time to first unscheduled contact with an asthma exacerbation**<br>HR 1.19 (95% CI 0.92 to 1.53) p=.185<br><br>Patient-reports<br>**Asthma-related QoL (AQ20)**<br>N: I=71 C=75 3 months: mean diff -2.56 (95% CI -3.89 to -1.24), p<.001 (favours I)<br>**Asthma-related self-efficacy**<br>N: I=152 C=160 3 months: mean diff 0.44 (95% CI 0.05 to 0.82), p=.027 (favours I)	Some evidence of effectiveness
Shah et al<br>2011<br>Australia	2-group RCT<br><br>Unclear	PACE initiative: develops skills for treating asthma, including supporting patients & families to self-manage<br><br>Standard care control	Implementation outcomes<br>**GP confidence in using 10 communication strategies**<br>1yr: Diff I-C: 22% (95% CI 3% to 40%) p=.03 (favours I) Numbers NR<br><br>**PRIMARY OUTCOME**<br>**Parent report: written asthma action plan received 1+ times**<br>1yr: I: 61% (of 101) C: 46% (of 104) diff 15% (95% CI 2% to 28%) p=.046<br>**GP report: written asthma action plan provided >70% of the time**<br>1yr: I: 76% (of 55) C: 53% (of 47) d 23% (95% CI 11% to 36%) p=.002<br>Parent report<br>**1+ hospital visits** 1yr I: 18% (or 101) C: 12% (of 106) diff 6% (95% CI -4% to 15%) p=.12<br>**ICS use** % diff I-C (95% CI)<br>Infrequent intermittent asthma (n=95): -24% (-43% to -5%) p=.03<br>Persistent asthma (n=21): 25% (-9% to 59%) p=.4<br>Frequent intermittent asthma (n=97): -3% (-23% to 17%) p=.9<br>**LABA use** % diff I-C (95% CI)<br>Infrequent intermittent asthma (n=95): -19% (-34% to -5%) p=.02<br>Persistent asthma (n=21) 28% (-11% to 66%) p=.4<br>Frequent intermittent asthma (n=97): -1% (-19% to 16%) p=1.0	Some evidence of effectiveness

Study, year, country	Design, risk of bias	Intervention summary	Key results	Conclusions
Cleland et al 2007<br>UK	2-group cluster RCT<br><br>Unclear	Focused on effective communication, self-management education for patient/families, and use of action plans<br><br>Standard care control	Implementation outcomes NR<br>Patient report, geometric mean (transformed 95% CI), numbers in each analysis NR<br>**PRIMARY OUTCOME**<br>**Asthma-related QoL (Mini AQLQ)** 6 months: I: 6.49 (6.40-6.59) C: 6.33 (6.23-6.44) p=.03 (favours I)<br>**Asthma control (ACQ)** 6 months: I: 3.14 (3.06-3.23) C: 3.2 (3.10-3.30) p=.43<br><br>Routine data, geometric mean (transformed 95% CI)<br>**Number of oral steroid courses** 6 months: I: 1.07 (1.04-1.10) C: 1.11 (1.07-1.45) p=.12<br>**Number of β2-agonist inhaler prescriptions**<br>6 months: I: 5.52 (5.02 to 6.08) C: 5.84 (5.20 to 6.55) p=.47	No evidence of effectiveness
Cohen et al 2014<br>Israel	5-group CCT<br><br>High	Focused on effective communication, self-management education for patient/families, and use of action plans<br><br>I1: GPs: Received: list of patients with uncontrolled asthma, request to meet with patients to consider ways of improving treatment, lecture on asthma treatment innovations<br><br>I2:GPs: I1 plus workshop<br><br>I3: GPs & nurses: I1 plus simulation training<br><br>I4: GPs & nurses: I1 plus I2 plus I3<br><br>Standard care control	Implementation outcomes NR<br>**PRIMARY (AND ONLY) OUTCOME**<br>**Change in rate of uncontrolled asthma** (based on inhaler purchase data)<br>Favours I for all<br>1yr-baseline<br>I1: 0.15 I2: 0.14 I3: 0.20 I4: 0.28 C: 0.003<br>Diff I1-C: 0.15 z-score 3.27 p<.01<br>Diff I2-C: 0.13 z-score 5.67 p<.01<br>Diff I3-C: 0.19 z-score 5.00 p<.01<br>Diff I4-C: 0.26 z-score 7.97 p<.01<br>2yr-1yr<br>I1: 0.07 I2: 0.09 I3: 0.07 I4: 0.08 C: 0.004<br>Diff I1-C: 0.06 z-score 1.70 p<.05<br>Diff I2-C: 0.09 z-score 4.17 p<.001<br>Diff I3-C: 0.06 z-score 2.35 p<.001<br>Diff I4-C: 0.08 z-score 3.13 p<.001	Some evidence of effectiveness
Evans et al 1997<br>USA	2-group cluster RCT<br><br>Unclear	Focused on effective communication, self-management education for patient/families, and use of action plans<br><br>Standard care control (but received guidelines, and supplies of same medications and delivery devices)	Primary outcome not specified; numbers in each analysis NR<br>Implementation outcomes<br>Caregiver-report, % (SE)<br>**Received education by physician on written treatment plan** 2yr: I: 78% (4) C: 76% (7) p=NS<br>**Received education by nurse on written treatment plan** 2yr: I: 60% (9) C: 53% (11) p=NS<br>**Received education by physician (Patient Education Index)** 2yr: I: 71% (3) C: 58% (7) p<.01<br>**Received education by nurse (Patient Education Index)** 2yr: I: 61% (4) C: 44% (9) p=NS<br>% patients (SE), based on dispensing database data	Some evidence of effectiveness

Study, year, country	Design, risk of bias	Intervention summary	Key results	Conclusions
			**Received any β-agonist** 1yr: I: 68% (4) C: 50% (7) p<.05 2yr: I: 74% (2) C: 52% (7) p<.05<br>**Received any inhaled anti-inflammatory**<br>1yr: I: 17% (3) C: 3% (3) p<.001 2yr: I: 25% (4) C: 2% (1) p<.001<br>**Received oral steroid** 1yr: I: 1% (1), C: 3% (3) p=NS 2yr: I: 5% (2) C: 1% (1) p=NS	
Homer et al<br>2005<br>USA	2-group cluster RCT<br><br>High	Learning collaborative project: participants identified performance gaps in their own practices' asthma care, and learning was based on these<br><br>Standard care control	All outcomes parent-reported; p-values for differences between groups in baseline vs 1yr comparisons<br>Implementation outcomes<br>**ONE OF PRIMARY OUTCOMES**<br>**Written asthma management plan received in past 12 months** 1yr: I: 54% C: 41% p=NS<br>**Hospitalization in past 12 months** 1yr: I: 2% C: 4% p=NS<br>**ED visit in past 12 months** 1yr: I: 17% C: 22% p=NS<br>**Asthma attack in past 12 months** 1yr: I: 40% C: 36% p=NS<br>**ONE OF PRIMARY OUTCOMES**<br>**Daily use of inhaled steroid medication in past 4 weeks** 1yr: I: 17% C: 17% p=NS<br>**ONE OF PRIMARY OUTCOMES**<br>**Daily use of controller medication in past 4 weeks** 1yr: I: 45% C: 39% p=NS	No evidence of effectiveness
Prabhakaran et al<br>2012<br>Singapore	3-group RCT<br><br>Unclear	Education covered general management of asthma, with self-management support being a specific topic addressed<br><br>I1: Workshop<br><br>I2: As I1 except lectures in e-learning format<br><br>I3: combination of I1 and I2	Primary outcome not specified<br>Implementation outcomes<br>**Knowledge**<br>Not assessed using a validated tool (significant improvement in knowledge from baseline to 3 months in all groups)<br>Patient outcomes NR	No evidence of effectiveness
Sheikh et al<br>2016<br>USA	2-group cluster RCT<br><br>Unclear	Education covered general management of asthma, with self-management support being a specific topic addressed<br><br>Control group received intervention for 6 months, after the initial 1yr follow-up	Primary outcome not specified<br>Implementation outcomes<br>(After both groups received intervention, self-reported whether noticed that treatment and/or knowledge changed: NS between groups: no comparison at 1yr when group 1 had intervention but group 2 had not)<br><br>Proportion of medical records with documentation of quality asthma care indicator at 1yr:<br>**Asthma education**: I: 56.1%, C: 19.5%, p≤.05<br>**Asthma action plan**: I: 29%, C: 5.4%, p≤.05<br>**Acute care visit**: I: 90.3%, C: 91.9%, NS	Some evidence of effectiveness

Study, year, country	Design, risk of bias	Intervention summary	Key results	Conclusions
			Patient outcomes NR	
Smeele et al<br>1999<br>Netherlands	2-group cluster RCT<br><br>Unclear	**FOCUS ON ASTHMA AND COPD**<br><br>Education covered general management of asthma, with self-management support being a specific topic addressed<br><br>Standard care control	Primary outcome not specified; includes asthma and COPD<br>Implementation outcomes<br>Mean change (95% CI) baseline to 1yr<br>**Knowledge score (0-10) on diagnosis and treatment of acute severe asthma and exacerbations**<br>1: +1.2 (0.0 to 2.4) C: -0.2 (-1.5 to 1.1) Diff +0.9 (-0.6 to 2.4) p=.25<br>Mean change (95% CI) baseline to 1yr, unit of analysis = GPs (16 I, 17 C)<br>**Patient report received written patient education**:<br>+3% (-7% to 13%) C: +7% (-1% to 15%) Diff -1% (-13% to 11%) p=.8<br>Change baseline to 1yr<br>**No. patients reporting symptom exacerbations past 3 months (ratio)**<br>I: 0 C: -0.11 p=.1	No evidence of effectiveness
Toelle et al<br>1993<br>Australia	2-group CCT<br><br>High	Focused on effective communication, self-management education for patient/families, and use of action plans<br><br>Standard care control	Primary outcome not specified<br>Implementation outcomes NR<br>Parent-report, past 6 months<br>**Unscheduled doctor/ ED visits**, mean (95% CI)<br>6 months: I: 1.51 (0.94 to 2.08) n=63 C: 1.67 (1.01 to 2.33) n=51 p=NS<br>**Symptoms limiting activity**, % (95% CI)<br>6 months: I: 18.60% (7.0% to 30.2%) n=43 C: 8.30% (0% to 17.3%) n=36 p=NS<br>**Parent knowledge of asthma score**, mean (95% CI)<br>6 months: I: 5.1 (4.64 to 5.56) n=63 C: 4.2 (3.71 to 4.69) n=51 p=NS<br>But FEV1 significantly higher in I than C, and bronchial responsiveness occurred at significantly higher threshold	No evidence of effectiveness
Tomson et al<br>1997<br>Sweden	2-group CCT<br><br>High	Education covered general management of asthma, with self-management support being a specific topic addressed<br><br>Standard care control	Primary outcome not specified<br>Implementation outcomes<br>**Advice to use PEF meter in a self-management plan provided**<br>GP-report, 1yr: I: 46% (of 44) C: 32% (of 19) p=.05<br>**Amount of inhaled steroids taken increased**<br>Patient-report: 18 months: I: 16% (of 138) C: 5% (of 19) p=NR<br>**Ratios of prescribed inhaled β-agonists to inhaled glucocorticoids**<br>measured as defined daily doses: p=NS for areas/'clusters'	No evidence of effectiveness
Volovitz et al<br>2003<br>Israel	4-group CCT<br><br>High	Education covered general management of asthma, with self-management support being a specific topic addressed	Primary outcome not specified; numbers in all analyses NR<br>Implementation outcomes<br>**Acute asthma intervention plan ownership**<br>Reported by sample of I patients: data NR in full so not extracted	No evidence of effectiveness

Study, year, country	Design, risk of bias	Intervention summary	Key results	Conclusions
		I1: Asthma education program; application of learning to future consultations (follow-up of patients according to protocol) monitored<br><br>I2: I1 except follow-up not monitored<br><br>C1: No intervention, patients in same region<br><br>C2: No intervention, patients in different region	**ICS/ LABA/ Leukotriene antagonist (Singulair)/ SABA use**<br>Based on database data; % diff baseline to 9 months, reported in figure: data NR in full so not extracted<br>Some indication that I groups more likely to increase use of controllers and decrease use of relievers than C groups, but not consistent<br>**Improvement in shortness of breath symptoms**<br>Patient report, I1: 64% (of 92) I2: 39% (of 100) p>.005 (significant)	

Note: ACQ = asthma control questionnaire; AQ20 = Airways Questionnaire 20; AQLQ = Asthma Quality of Life Questionnaire; CCT = controlled clinical trial; C = control group; CI = confidence interval; COPD = chronic obstructive pulmonary disease; diff = difference; ED = emergency department; FEV1 = forced expiratory volume in one second; GP = general practitioner; HCP=healthcare professional; HR = hazard ratio; I = intervention group; ICS = inhaled corticosteroid; LABA = long-acting β-agonist; NR = not reported; NS = not significant; OR = odds ratio; PACE = Physician Asthma Care Education; PEF = peak expiratory flow; QoL = quality of life; RCT = randomized controlled trial; REF = reference category for categorical predictor (e.g. control vs. intervention); SABA = short-acting β-agonist; SD = standard deviation

Online supplementary file 4: Grey Literature Review Summary

Methods

We conducted a review of grey literature, informed by published methodological guidance.[1] Eligibility criteria: The target population was healthcare professionals providing care to people with asthma. We included any program designed to educate practice teams and/or professionals in delivering education or supported self-management to patients with asthma. Eligible outcomes were primary, secondary, and process outcomes defined in the systematic review, as well as NHS Education for Scotland Outcomes: engagement, education, performance, and service. Relevant materials published in English since 2010 were included. Research conducted in any healthcare setting was of interest, but initiatives implemented within primary care teams were of particular interest.

Searching and screening: Three search strategies were used: database searching (conducted as part of the systematic review), customized Google searches, and targeted websites via consultation with contact experts. Authors were contacted for information if necessary. Screening was completed by one reviewer (AB).

Risk of bias assessment: Studies were assessed for quality using a checklist for grey literature (AACODS checklist) which focuses on Authority, Accuracy, Coverage, Objectivity, Date, and Significance.[2]

Data extraction and synthesis: Data were extracted and synthesized by one reviewer (AB) using the same data extraction form and analysis frameworks as described for the systematic review.

Results

In total, 744 records were retrieved and screened (Figure S1). Three studies were included.[3-5] The studies were heterogeneous, and few methodological details were reported. Two studies had moderate or severe risk of bias. Reports tended to highlight the positive, with all studies reporting some evidence of effectiveness. This in conjunction with the small number of studies, made it difficult to distinguish features of effective/ineffective programs. Face-to-face lectures were the only tool commonly used to deliver educational content. All studies provided at least partial information about how the education was developed.[3-5] One used the Plan, Do, Study, Act (PDSA) rapid cycles of change approach.[4] Two described involving stakeholders (healthcare professionals and/or patients) in the development of the education.[3,5]

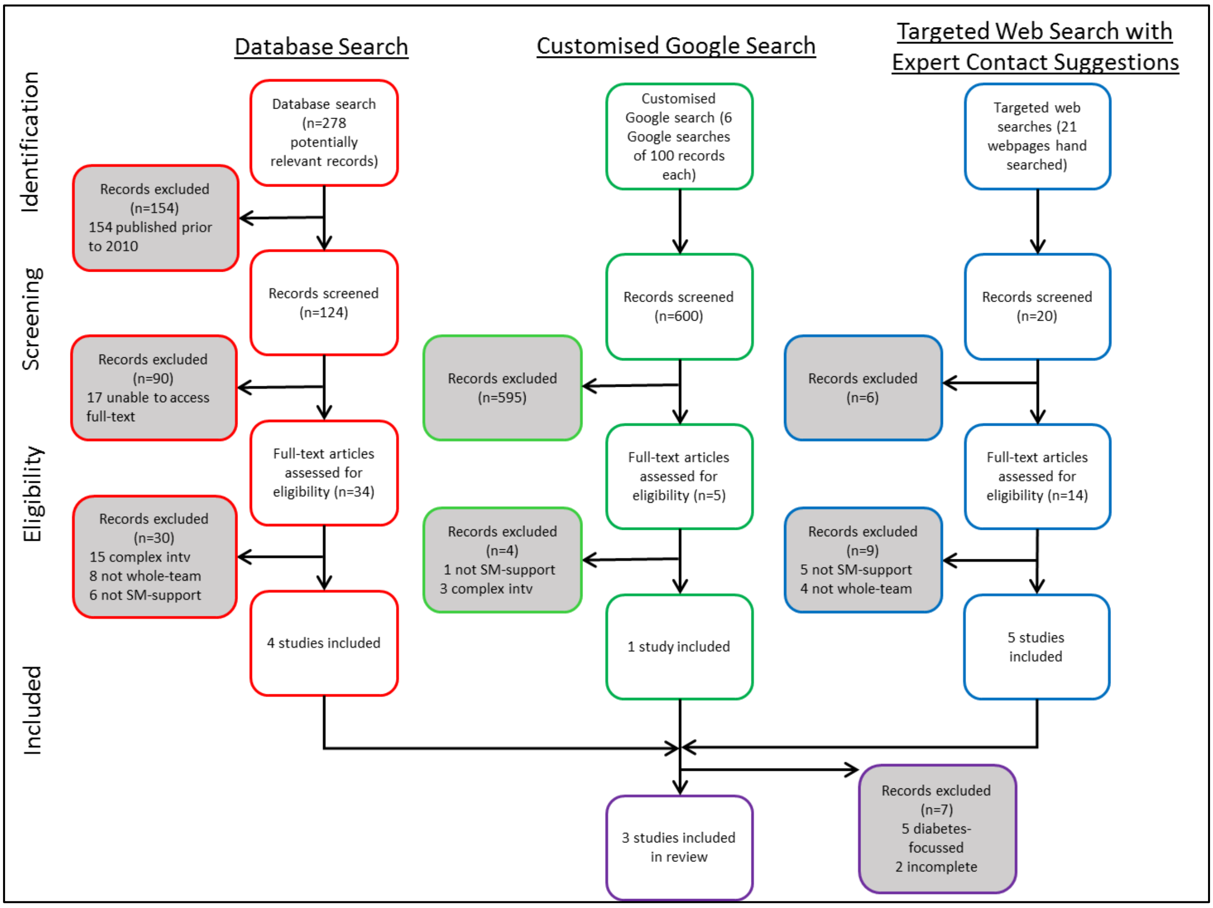

Figure S1. Adapted PRISMA flow diagram for grey literature search for studies evaluating education for professionals implementing supported self-management for asthma or diabetes

Note: Searches included interventions reporting educational initiatives related to asthma and diabetes; studies separated at full text screening and synthesised separately.

References

1. Godin K, Stapleton J, Kirkpatrick SI, Hanning RM, Leatherdale ST. Applying systematic review search methods to the grey literature: a case study examining guidelines for school-based breakfast programs in Canada. *Systematic Reviews* 2015; **4**(1): 138.
2. Tyndall J. How low can you go? Towards a hierarchy of grey literature. *Dreaming 08 – Australian Library and Information Association Biennial Conference 2 – 5 September 2008 Alice Springs Convention Centre, Alice Springs, NT Australia* 2008.
3. Bender BG, Dickinson P, Rankin A, Wamboldt FS, Zittleman L, Westfall JM. The Colorado Asthma Toolkit Program: A Practice Coaching Intervention from the High Plains Research Network. *The Journal of the American Board of Family Medicine* 2011; **24**(3): 240-8.
4. Chandler L. Improving adult asthma care: Emerging learning from the national improvement projects. *NHS Improvement-Lung* 2016; **1**: 12-3.
5. Kaferle JE, Wimsatt LA. A Team-Based Approach to Providing Asthma Action Plans. *The Journal of the American Board of Family Medicine* 2012; **25**(2): 247-9.

Online supplementary file 5: Search Strategies

MEDLINE

1	Primary Health Care/ or Family Practice/ or General Practice/
2	(primary care or primary medical care or primary health care or primary healthcare or general practice or family practice).mp.
3	Health Personnel/ or Medical Staff/
4	((healthcare or health care) adj3 (provider? or practitioner? or professional?)).mp.
5	Physicians/ or Physicians, Primary Care/ or Physicians, Family/ or General Practitioners/
6	(general practitioner? or medical practitioner? or physician? or clinician? or doctor? or GP?).mp.
7	Nurses/ or Nursing Staff/ or Nurse Practitioners/ or Family Nurse Practitioners/
8	(nurs* or practice nurs* or community nurs* or nurs* practitioner?).mp.
9	Medical Secretaries/ or Medical Receptionists/ or Health Facility Administrators/ or Administrative Personnel/
10	(secretar* or reception* or admin*).mp.
11	Pharmacists/
12	pharmacist?.mp.
13	Health Educators/
14	health educator?.mp.
15	Patient Care Team/
16	((primary care or primary care practice or health care or healthcare or medical care or patient care or care or general practice or family practice) adj3 team*).mp.
17	or/1-16
18	Education/ or Health Education/
19	(educat* or train*).mp.
20	(skill? adj3 develop*).mp.
21	Education, Professional/ or Education, Continuing/ or Staff Development/
22	(professional development or CPD).mp.
23	((interprofessional or inter professional or inter-professional) adj3 (educat* or train* or develop* or skill?)).mp.
24	((team? or group?) adj3 (educat* or train* or develop* or skill?)).mp.
25	Education, Medical/ or Education, Medical, Continuing/
26	(continuing medical education or CME).mp.
27	Education, Nursing/ or Education, Nursing, Continuing/ or Nursing Education Research/ or Nursing Evaluation Research/
28	Education, Pharmacy/ or Education, Pharmacy, Continuing/
29	Quality Improvement/
30	(quality adj3 improv*).mp.
31	or/18-30
32	Disease Management/
33	disease management.mp.
34	Self Care/ or Self Administration/ or Self Medication/
35	(self-manag* or selfmanag* or self-car* or selfcar* or self-help or selfhelp or self-administrat* or selfadministrat* or self-monitor* or selfmonitor* or self-medicat* or selfmedicat*).mp.
36	(self adj3 (manag* or car* or help or administrat* or monitor* or medicat*)).mp.
37	Quality of Health Care/
38	(quality adj3 (care or healthcare or health care)).mp.
39	Professional-Patient Relations/ or Physician-Patient Relations/ or Nurse-Patient Relations/
40	(patient? adj3 (relat* or communicat*)).mp.
41	((action or treat* or car* or written or manag* or medicat*) adj3 plan*).mp.
42	((self-manag* or self manag* or selfmanag* or self-car* or self car* or selfcar* or self-help or self help or selfhelp or self-administrat* or self administrat* or selfadministrat* or self-monitor* or self monitor* or selfmonitor* or self-medicat* or self medicat* or selfmedicat* or self-treat* or self treat* or selftreat*) adj3 plan*).mp.
43	(exacerbat* or attack?).mp.
44	asthma control test.mp.
45	Hospitalization/
46	hospitali?ation?.mp.
47	After-Hours Care/
48	(out of hours or out-of-hours or OOH).mp.
49	Office Visits/
50	((office or hospital or emergency department or ED or A&E or A & E or "accident and emergency") adj3 (visit* or refer* or admission?)).mp.
51	((care or service?) adj3 (utili?ation or use?)).mp.
52	Patient Education/
53	Blood Glucose Self Monitoring/
54	Hemoglobin A, Glycosylated/
55	HbA1c.mp.
56	Hypoglycemia/ or Hyperglycemia/
57	Diabetic Ketoacidosis/ or Hyperglycemic Hyperosmolar Nonketotic Coma/ or Diabetic Coma/

58	(hyperosmolar hyperglyc?emic nonketotic syndrome or DKA or HNNS or HONK).mp.
59	glyc?emic control.mp.
60	or/32-59
61	Asthma/
62	(asthma or wheez*).mp.
63	(antiasthma* or anti-asthma*).mp.
64	Respiratory Hypersensitivity/
65	((bronchial* or respiratory or airway* or lung*) adj3 (hypersensitiv* or hyperreactiv* or allerg* or insufficiency)).mp.
66	Bronchial Spasm/
67	Bronchoconstriction/
68	(bronch* adj3 (constrict* or spas*)).mp.
69	(bronchoconstrict* or bronchospas*).mp.
70	bronchial hyperreactivity.mp.
71	respiratory sounds.mp.
72	Diabetes Mellitus/
73	diabet*.mp.
74	Diabetes Mellitus, Type 1/
75	((diabet* or dm) adj5 (typ* adj3 (one or "1" or I))).mp.
76	Diabetes Mellitus, Type 2/
77	((diabet* or dm) adj5 (typ* adj3 (two or "2" or II))).mp.
78	Insulin Resistance/
79	((insulin or noninsulin or non-insulin) adj3 (resistan* or depend*)).mp.
80	(DM or DM1 or DM2 or T1D or T1DM or T2D or T2DM or NIDDM or IDDM or MODY).mp.
81	Glucose Intolerance/
82	glucose $tolerance.mp.
83	or/61-82
84	Pragmatic Clinical Trial/ or Clinical Trial/ or Randomized Controlled Trial/ or Controlled Clinical Trial/
85	randomi?ed controlled trial.pt.
86	controlled clinical trial.pt.
87	clinical trial.pt.
88	pragmatic clinical trial.mp.
89	(randomi?ed or randomly).ti,ab.
90	trial.ti,ab.
91	group?.ti,ab.
92	or/84-91
93	17 and 31 and 60 and 83 and 92
94	(review or comment or editorial).pt.
95	93 not 94
96	(Animals/ or Nonhuman/) not Humans/
97	95 not 96
98	limit 97 to yr="1990 -Current"

EMBASE

1	primary health care/ or primary medical care/ or general practice/
2	(primary care or primary medical care or primary health care or primary healthcare or general practice or family practice).mp.
3	health care personnel/ or medical staff/
4	((healthcare or health care) adj3 (provider? or practitioner? or professional?)).mp.
5	physician/ or general practitioner/
6	(general practitioner? or medical practitioner? or physician? or clinician? or doctor? or gp?).mp.
7	nurse/ or nursing staff/ or nurse practitioner/ or family nurse practitioner/
8	(nurs* or practice nurs* or community nurs* or nurs* practitioner?).mp.
9	administrative personnel/
10	(secretar* or reception* or admin*).mp.
11	pharmacist/
12	pharmacist?.mp.
13	health educator/
14	health educator?.mp.
15	((primary care or primary care practice or health care or healthcare or medical care or patient care or care or general practice or family practice) adj3 team*).mp.
16	or/1-15
17	education/ or health education/ or training/
18	(educat* or train*).mp.
19	(skill? adj3 develop*).mp.
20	continuing education/ or staff training/
21	(professional development or cpd).mp.
22	((interprofessional or inter professional or inter-professional) adj3 (educat* or train* or develop* or skill?)).mp.
23	((team? or group?) adj3 (educat* or train* or develop* or skill?)).mp.
24	clinical education/ or medical education/
25	(continuing medical education or CME).mp.
26	nurse education/ or nurse training/ or nursing evaluation research/
27	(quality adj3 improv*).mp.
28	or/17-27
29	disease management/
30	disease management.mp.
31	self care/ or drug self administration/ or self medication/ or self monitoring/
32	(self-manag* or selfmanag* or self-car* or selfcar* or self-help or selfhelp or self-administrat* or selfadministrat* or self-monitor* or selfmonitor* or self-medicat* or selfmedicat*).mp.
33	(self adj3 (manag* or car* or help or administrat* or monitor* or medicat*)).mp.
34	health care quality/
35	(quality adj3 (care or healthcare or health care)).mp.
36	doctor patient relation/ or nurse patient relationship/
37	(patient? adj3 (relat* or communicat*)).mp.
38	((action or treat* or car* or written or manag* or medicat*) adj3 plan*).mp.
39	((self-manag* or self manag* or selfmanag* or self-car* or self car* or selfcar* or self-help or self help or selfhelp or self-administrat* or self administrat* or selfadministrat* or self-monitor* or self monitor* or selfmonitor* or self-medicat* or self medicat* or selfmedicat* or self-treat* or self treat* or selftreat*) adj3 plan*).mp.
40	(exacerbat* or attack?).mp.
41	asthma control test.mp.
42	hospitalization/ or hospital admission/
43	hospitali?ation?.mp.
44	(out of hours or out-of-hours or OOH).mp.
45	((office or hospital or emergency department or ED or A&E or A & E or "accident and emergency") adj3 (visit* or refer* or admission?)).mp.
46	((care or service?) adj3 (utili?ation or use?)).mp.
47	patient education/
48	diabetes education/
49	blood glucose monitoring/
50	glycosylated hemoglobin/ or hemoglobin a1c/
51	HbA1c.mp.
52	hypoglycemia/ or insulin hypoglycemia/ or hyperglycemia/
53	diabetic ketoacidosis/ or nonketotic diabetic coma/ or diabetic coma/
54	(hyperosmolar hyperglyc?emic nonketotic syndrome or DKA or HNNS or HONK).mp.
55	glycemic control/
56	glyc?emic control.mp.
57	or/29-56
58	asthma/
59	(asthma or wheez*).mp.
60	(antiasthma* or anti-asthma*).mp.

61	((bronchial* or respiratory or airway* or lung*) adj3 (hypersensitiv* or hyperreactiv* or allerg* or insufficiency)).mp.
62	bronchospasm/
63	bronchoconstriction/
64	(bronch* adj3 (constrict* or spas*)).mp.
65	(bronchoconstrict* or bronchospas*).mp.
66	bronchial hyperreactivity.mp.
67	respiratory sounds.mp.
68	diabetes mellitus/
69	diabet*.mp.
70	((diabet* or dm) adj5 (typ* adj3 (one or "1" or I))).mp.
71	((diabet* or dm) adj5 (typ* adj3 (two or "2" or II))).mp.
72	insulin resistance/
73	((insulin or noninsulin or non-insulin) adj3 (resistan* or depend*)).mp.
74	(DM or DM1 or DM2 or T1D or T1DM or T2D or T2DM or NIDDM or IDDM or MODY).mp.
75	glucose tolerance/ or glucose intolerance/
76	glucose $tolerance.mp.
77	or/58-76
78	controlled clinical trial/ or clinical trial/ or randomized controlled trial/
79	pragmatic clinical trial.mp.
80	randomi?ed controlled trial.mp.
81	controlled clinical trial.mp.
82	clinical trial.mp.
83	(randomi?ed or randomly).ti,ab.
84	trial.ti,ab.
85	group?.ti,ab.
86	or/78-85
87	16 and 28 and 57 and 77 and 86
88	(review or comment or editorial).pt.
89	87 not 88
90	(animal/ or nonhuman/) not human/
91	89 not 90
92	limit 91 to yr="1990 -Current"
93	limit 92 to embase

PsycINFO

1	Primary Health Care/ or Family Medicine/
2	(primary care or primary medical care or primary health care or primary healthcare or general practice or family practice).mp.
3	Health Personnel/ or Medical Personnel/
4	((healthcare or health care) adj3 (provider? or practitioner? or professional?)).mp.
5	Physicians/ or Clinicians/ or Family Physicians/ or General Practitioners/
6	(general practitioner? or medical practitioner? or physician? or clinician? or doctor? or GP?).mp.
7	Nurses/
8	(nurs* or practice nurs* or community nurs* or nurs* practitioner?).mp.
9	Secretarial Personnel/ or Clerical Personnel/
10	(secretar* or reception* or admin*).mp.
11	Pharmacists/
12	pharmacist?.mp.
13	health educator?.mp.
14	((primary care or primary care practice or health care or healthcare or medical care or patient care or care or general practice or family practice) adj3 team*).mp.
15	or/1-14
16	Education/ or Health Education/
17	Training/ or Personnel Training/
18	(educat* or train*).mp.
19	(skill? adj3 develop*).mp.
20	Professional Development/ or Continuing Education/
21	(professional development or CPD).mp.
22	((interprofessional or inter professional or inter-professional) adj3 (educat* or train* or develop* or skill?)).mp.
23	((team? or group?) adj3 (educat* or train* or develop* or skill?)).mp.
24	Medical Education/
25	(continuing medical education or CME).mp.
26	Nursing Education/
27	(quality adj3 improv*).mp.
28	or/16-27
29	Disease Management/
30	disease management.mp.
31	Self Medication/ or Drug Self Administration/ or Self Monitoring/ or Self Management/ or Self Care Skills/
32	(self-manag* or selfmanag* or self-car* or selfcar* or self-help or selfhelp or self-administrat* or selfadministrat* or self-monitor* or selfmonitor* or self-medicat* or selfmedicat*).mp.
33	(self adj3 (manag* or car* or help or administrat* or monitor* or medicat*)).mp.
34	Quality of Care/
35	(quality adj3 (care or healthcare or health care)).mp.
36	(patient? adj3 (relat* or communicat*)).mp.
37	((action or treat* or car* or written or manag* or medicat*) adj3 plan*).mp.
38	((self-manag* or self manag* or selfmanag* or self-car* or self car* or selfcar* or self-help or self help or selfhelp or self-administrat* or self administrat* or selfadministrat* or self-monitor* or self monitor* or selfmonitor* or self-medicat* or self medicat* or selfmedicat* or self-treat* or self treat* or selftreat*) adj3 plan*).mp.
39	(exacerbat* or attack?).mp.
40	asthma control test.mp.
41	Hospitalization/ or Hospital Admission/
42	hospitali?ation?.mp.
43	(out of hours or out-of-hours or OOH).mp.
44	((office or hospital or emergency department or ED or A&E or A & E or "accident and emergency") adj3 (visit* or refer* or admission?)).mp.
45	((care or service?) adj3 (utili?ation or use?)).mp.
46	Client Education/
47	blood glucose self monitoring.mp.
48	HbA1c.mp.
49	Hypoglycemia/ or Hyperglycemia/
50	(diabetic ketoacidosis or hyperglycemic hyperosmolar nonketotic coma or diabetic coma).mp.
51	(hyperosmolar hyperglyc?emic nonketotic syndrome or DKA or HNNS or HONK).mp.
52	glyc?emic control.mp.
53	or/29-52
54	Asthma/
55	(asthma or wheez*).mp.
56	(antiasthma* or anti-asthma*).mp.
57	((bronchial* or respiratory or airway* or lung*) adj3 (hypersensitiv* or hyperreactiv* or allerg* or insufficiency)).mp.
58	(bronch* adj3 (constrict* or spas*)).mp.
59	(bronchoconstrict* or bronchospas*).mp.
60	bronchial hyperreactivity.mp.

61	respiratory sounds.mp.
62	Diabetes Mellitus/ or Diabetes/
63	diabet*.mp.
64	((diabet* or dm) adj5 (typ* adj3 (one or "1" or I))).mp.
65	((diabet* or dm) adj5 (typ* adj3 (two or "2" or II))).mp.
66	((insulin or noninsulin or non-insulin) adj3 (resistan* or depend*)).mp.
67	(DM or DM1 or DM2 or T1D or T1DM or T2D or T2DM or NIDDM or IDDM or MODY).mp.
68	glucose $tolerance.mp.
69	or/54-68
70	Clinical Trials/
71	(pragmatic clinical trial or randomi?ed controlled trial or controlled clinical trial or clinical trial).mp.
72	(randomi?ed or randomly).ti,ab.
73	trial.ti,ab.
74	group?.ti,ab.
75	or/70-74
76	15 and 28 and 53 and 69 and 75
77	(Animals/ or Nonhuman/) not Humans/
78	76 not 77
79	limit 78 to yr="1990 -Current"

AMED

1	Primary Health Care/ or Family Practice/
2	(primary care or primary medical care or primary health care or primary healthcare or general practice or family practice).mp.
3	Health Personnel/
4	((healthcare or health care) adj3 (provider? or practitioner? or professional?)).mp.
5	Physicians/
6	(general practitioner? or medical practitioner? or physician? or clinician? or doctor? or GP?).mp.
7	Nurses/ or Nursing Staff/
8	(nurs* or practice nurs* or community nurs* or nurs* practitioner?).mp.
9	Administrative Personnel/
10	(secretar* or reception* or admin*).mp.
11	pharmacist?.mp.
12	health educator?.mp.
13	Patient Care Team/
14	((primary care or primary care practice or health care or healthcare or medical care or patient care or care or general practice or family practice) adj3 team*).mp.
15	or/1-14
16	Education/ or Health Education/
17	(educat* or train*).mp.
18	(skill? adj3 develop*).mp.
19	Education Professional/ or Education Continuing/
20	Staff Development/
21	(professional development or CPD).mp.
22	((interprofessional or inter professional or inter-professional) adj3 (educat* or train* or develop* or skill?)).mp.
23	((team? or group?) adj3 (educat* or train* or develop* or skill?)).mp.
24	Education Medical/
25	(continuing medical education or CME).mp.
26	Education Nursing/
27	(quality adj3 improv*).mp.
28	or/16-27
29	Disease Management/
30	disease management.mp.
31	Self Care/
32	(self-manag* or selfmanag* or self-car* or selfcar* or self-help or selfhelp or self-administrat* or selfadministrat* or self-monitor* or selfmonitor* or self-medicat* or selfmedicat*).mp.
33	(self adj3 (manag* or car* or help or administrat* or monitor* or medicat*)).mp.
34	Quality of Health Care/
35	(quality adj3 (care or healthcare or health care)).mp.
36	Professional Patient Relations/ or Physician Patient Relations/ or Nurse Patient Relations/
37	(patient? adj3 (relat* or communicat*)).mp.
38	((action or treat* or car* or written or manag* or medicat*) adj3 plan*).mp.
39	((self-manag* or self manag* or selfmanag* or self-car* or self car* or selfcar* or self-help or self help or selfhelp or self-administrat* or self administrat* or selfadministrat* or self-monitor* or self monitor* or selfmonitor* or self-medicat* or self medicat* or selfmedicat* or self-treat* or self treat* or selftreat*) adj3 plan*).mp.
40	(exacerbat* or attack?).mp.
41	asthma control test.mp.
42	Hospitalization/
43	hospitali?ation?.mp.
44	After Hours Care/
45	(out of hours or out-of-hours or OOH).mp.
46	((office or hospital or emergency department or ED or A&E or A & E or "accident and emergency") adj3 (visit* or refer* or admission?)).mp.
47	((care or service?) adj3 (utili?ation or use?)).mp.
48	Patient Education/
49	blood glucose self monitoring.mp.
50	HbA1c.mp.
51	Hypoglycemia/ or Hyperglycemia/
52	(diabetic ketoacidosis or hyperglyc?emic hyperosmolar nonketotic coma or diabetic coma).mp.
53	(hyperosmolar hyperglyc?emic nonketotic syndrome or DKA or HNNS or HONK).mp.
54	glyc?emic control.mp.
55	or/29-54
56	Asthma/
57	(asthma or wheez*).mp.
58	(antiasthma* or anti-asthma*).mp.
59	Respiratory Hypersensitivity/
60	((bronchial* or respiratory or airway* or lung*) adj3 (hypersensitiv* or hyperreactiv* or allerg* or insufficiency)).mp.

61	(bronch* adj3 (constrict* or spas*)).mp.
62	(bronchoconstrict* or bronchospas*).mp.
63	bronchial hyperreactivity.mp.
64	respiratory sounds.mp.
65	Diabetes Mellitus/
66	diabet*.mp.
67	Diabetes Mellitus Type 1/
68	((diabet* or dm) adj5 (typ* adj3 (one or "1" or I))).mp.
69	Diabetes Mellitus Type 2/
70	((diabet* or dm) adj5 (typ* adj3 (two or "2" or II))).mp.
71	Insulin Resistance/
72	((insulin or noninsulin or non-insulin) adj3 (resistan* or depend*)).mp.
73	(DM or DM1 or DM2 or T1D or T1DM or T2D or T2DM or NIDDM or IDDM or MODY).mp.
74	glucose $tolerance.mp.
75	or/56-74
76	Clinical Trials/ or Randomized Controlled Trials/
77	pragmatic clinical trial.mp.
78	randomi?ed controlled trial.pt.
79	controlled clinical trial.pt.
80	clinical trial.pt.
81	(randomi?ed or randomly).ti,ab.
82	trial.ti,ab.
83	group?.ti,ab.
84	or/76-83
85	15 and 28 and 55 and 75 and 84
86	(letter or review or comment or editorial).pt.
87	85 not 86
88	(Animals/ or Nonhuman/) not Humans/
89	87 not 88
90	limit 89 to yr="1990 -Current"

Global Health

1	Primary Health Care/
2	(primary care or primary medical care or primary health care or primary healthcare or general practice or family practice).mp.
3	Health Care Workers/
4	((healthcare or health care) adj3 (provider? or practitioner? or professional?)).mp.
5	Physicians/ or General Practitioners/
6	(general practitioner? or medical practitioner? or physician? or clinician? or doctor? or GP?).mp.
7	Nurses/
8	(nurs* or practice nurs* or community nurs* or nurs* practitioner?).mp.
9	(secretar* or reception* or admin*).mp.
10	pharmacist?.mp.
11	health educator?.mp.
12	((primary care or primary care practice or health care or healthcare or medical care or patient care or care or general practice or family practice) adj3 team*).mp.
13	or/1-12
14	Education/ or Health Education/
15	Training/
16	(educat* or train*).mp.
17	(skill? adj3 develop*).mp.
18	Professional Education/ or Continuing Education/
19	(professional development or CPD).mp.
20	((interprofessional or inter professional or inter-professional) adj3 (educat* or train* or develop* or skill?)).mp.
21	((team? or group?) adj3 (educat* or train* or develop* or skill?)).mp.
22	Medical Education/
23	(continuing medical education or CME).mp.
24	(quality adj3 improv*).mp.
25	or/14-24
26	disease management.mp.
27	Self Care/ or Self Management/
28	(self-manag* or selfmanag* or self-car* or selfcar* or self-help or selfhelp or self-administrat* or selfadministrat* or self-monitor* or selfmonitor* or self-medicat* or selfmedicat*).mp.
29	(self adj3 (manag* or car* or help or administrat* or monitor* or medicat*)).mp.
30	Quality of Care/
31	(quality adj3 (care or healthcare or health care)).mp.
32	(patient? adj3 (relat* or communicat*)).mp.
33	((action or treat* or car* or written or manag* or medicat*) adj3 plan*).mp.
34	((self-manag* or self manag* or selfmanag* or self-car* or self car* or selfcar* or self-help or self help or selfhelp or self-administrat* or self administrat* or selfadministrat* or self-monitor* or self monitor* or selfmonitor* or self-medicat* or self medicat* or selfmedicat* or self-treat* or self treat* or selftreat*) adj3 plan*).mp.
35	(exacerbat* or attack?).mp.
36	asthma control test.mp.
37	Hospital Admission/ or Hospital Stay/
38	hospitali?ation?.mp.
39	(out of hours or out-of-hours or OOH).mp.
40	((office or hospital or emergency department or ED or A&E or A & E or "accident and emergency") adj3 (visit* or refer* or admission?)).mp.
41	((care or service?) adj3 (utili?ation or use?)).mp.
42	Patient Education/
43	blood glucose self monitoring.mp.
44	Haemoglobin A1/
45	HbA1c.mp.
46	Hypoglycaemia/ or Hyperglycaemia/
47	Ketoacidosis/ or Diabetic Coma/
48	hyperglyc?emic hyperosmolar nonketotic coma.mp.
49	(hyperosmolar hyperglyc?emic nonketotic syndrome or DKA or HNNS or HONK).mp.
50	glyc?emic control.mp.
51	or/26-50
52	Asthma/ or Bronchial Asthma/
53	(asthma or wheez*).mp.
54	(antiasthma* or anti-asthma*).mp.
55	Respiratory Hypersensitivity/
56	((bronchial* or respiratory or airway* or lung*) adj3 (hypersensitiv* or hyperreactiv* or allerg* or insufficiency)).mp.
57	(bronch* adj3 (constrict* or spas*)).mp.
58	(bronchoconstrict* or bronchospas*).mp.
59	bronchial hyperreactivity.mp.
60	respiratory sounds.mp.

61	Diabetes Mellitus/ or Diabetes/
62	diabet*.mp.
63	Type 1 Diabetes/
64	((diabet* or dm) adj5 (typ* adj3 (one or "1" or I))).mp.
65	Type 2 Diabetes/
66	((diabet* or dm) adj5 (typ* adj3 (two or "2" or II))).mp.
67	Insulin Resistance/
68	((insulin or noninsulin or non-insulin) adj3 (resistan* or depend*)).mp.
69	(DM or DM1 or DM2 or T1D or T1DM or T2D or T2DM or NIDDM or IDDM or MODY).mp.
70	Glucose Tolerance/
71	glucose $tolerance.mp.
72	or/52-71
73	Clinical Trials/ or Randomized Controlled Trials/
74	pragmatic clinical trial.mp.
75	randomi?ed controlled trial.mp.
76	controlled clinical trial.mp.
77	clinical trial.mp.
78	(randomi?ed or randomly).ti,ab.
79	trial.ti,ab.
80	group?.ti,ab.
81	or/73-80
82	13 and 25 and 51 and 72 and 81
83	(review or comment or editorial).pt.
84	82 not 83
85	(Animals/ or Nonhuman/) not Humans/
86	84 not 85
87	limit 86 to yr="1990 -Current"

CINAHL Plus

S108	S106 NOT S107
S107	(MH "Animals")
S106	S104 NOT S105
S105	PT review or comment or editorial
S104	S23 AND S39 AND S68 AND S91 AND S103
S103	S92 OR S93 OR S94 OR S95 OR S96 OR S97 OR S98 OR S99 OR S100 OR S101 OR S102
S102	AB group*
S101	TI group*
S100	AB trial
S99	TI trial
S98	AB randomi*ed or randomly
S97	TI randomi*ed or randomly
S96	MW controlled clinical trial or pragmatic clinical trial
S95	AB controlled clinical trial or pragmatic clinical trial
S94	TI controlled clinical trial or pragmatic clinical trial
S93	PT randomi*ed controlled trial or clinical trial
S92	(MH "Clinical Trials") OR (MH "Randomized Controlled Trials")
S91	S69 OR S70 OR S71 OR S72 OR S73 OR S74 OR S75 OR S76 OR S77 OR S78 OR S79 OR S80 OR S81 OR S82 OR S83 OR S84 OR S85 OR S86 OR S87 OR S88 OR S89 OR S90
S90	TX glucose *tolerance
S89	(MH "Glucose Intolerance")
S88	TX DM or DM1 or DM2 or T1D or T1DM or T2D or T2DM or NIDDM or IDDM or MODY
S87	TX (insulin or noninsulin or non-insulin) N3 (resistan* or depend*)
S86	(MH "Insulin Resistance")
S85	TX (diabet* or dm) N5 (typ* N3 (two or "2" or II))
S84	(MH "Diabetes Mellitus, Type 2")
S83	TX (diabet* or dm) N5 (typ* N3 (one or "1" or I))
S82	(MH "Diabetes Mellitus, Type 1")
S81	TX diabet*
S80	(MH "Diabetes Mellitus")
S79	TX respiratory sounds
S78	TX bronchial hyperreactivity
S77	TX bronchoconstrict* or bronchospas*
S76	TX bronch* N3 (constrict* or spas*)
S75	(MH "Bronchoconstriction")
S74	(MH "Bronchial Spasm")
S73	TX (bronchial* or respiratory or airway* or lung*) N3 (hypersensitiv* or hyperreactiv* or allerg* or insufficiency)
S72	(MH "Respiratory Hypersensitivity")
S71	TX antiashtma* or anti-asthma*
S70	TX asthma or wheez*
S69	(MH "Asthma")
S68	S40 OR S41 OR S42 OR S43 OR S44 OR S45 OR S46 OR S47 OR S48 OR S49 OR S50 OR S51 OR S52 OR S53 OR S54 OR S55 OR S56 OR S57 OR S58 OR S59 OR S60 OR S61 OR S62 OR S63 OR S64 OR S65 OR S66 OR S67
S67	TX glyc*emic control
S66	(MH "Glycemic Control")
S65	TX hyperosmolar hyperglyc*emic nonketotic syndrome or DKA or HNNS or HONK
S64	(MH "Diabetic Ketoacidosis") OR (MH "Diabetic Coma") OR (MH "Hyperglycemic Hyperosmolar Nonketotic Coma")
S63	(MH "Hypoglycemia") OR (MH "Hyperglycemia")
S62	TX HbA1c
S61	(MH "Hemoglobin A, Glycosylated")
S60	(MH "Blood Glucose Self-Monitoring")
S59	(MH "Patient Education") OR (MH "Diabetes Education")
S58	TX (care or service*) N3 (utili*ation or use*)
S57	TX ("office" or "hospital" or "emergency department" or "ED" or "A&E" or "A & E" or "accident and emergency") N3 (visit* or refer* or admission*)
S56	(MH "Office Visits")
S55	TX out of hours or out-of-hours or OOH
S54	TX hospitali*ation*
S53	(MH "Hospitalization")
S52	TX asthma control test
S51	TX exacerbat* or attack*
S50	TX (self-manag* or self manag* or selfmanag* or self-car* or self car* or selfcar* or self-help or self help or selfhelp or self-administrat* or self administrat* or selfadministrat* or self-monitor* or self monitor* or selfmonitor* or self-medicat* or self medicat* or selfmedicat* or self-treat* or self treat* or selftreat*) N3 plan*
S49	TX (action or treat* or car* or written or manag* or medicat*) N3 plan*

S48	TX patient N3 (relat* or communicat*)
S47	(MH "Professional-Patient Relations") OR (MH "Physician-Patient Relations") OR (MH "Nurse-Patient Relations")
S46	TX quality N3 (care or healthcare or health care)
S45	(MH "Quality of Health Care")
S44	TX self N3 (manag* or car* or help or administrat* or monitor* or medicat*)
S43	TX self-manag* or selfmanag* or self-car* or selfcar* or self-help or selfhelp or self-administrat* or selfadministrat* or self-monitor* or selfmonitor* or self-medicat* or selfmedicat*
S42	(MH "Self Care") OR (MH "Self Administration") OR (MH "Self Medication")
S41	TX disease management
S40	(MH "Disease Management")
S39	S24 OR S25 OR S26 OR S27 OR S28 OR S29 OR S30 OR S31 OR S32 OR S33 OR S34 OR S35 OR S36 OR S37 OR S38
S38	TX quality N3 improv*
S37	(MH "Quality Improvement") OR (MH "Evaluation and Quality Improvement Program")
S36	(MH "Education, Pharmacy")
S35	(MH "Education, Nursing") OR (MH "Education, Nursing, Continuing") OR (MH "Education, Nursing, Research-Based")
S34	TX continuing medical education or CME
S33	(MH "Education, Medical") OR (MH "Education, Medical, Continuing") OR (MH "Education, Clinical")
S32	(MH "Staff Development")
S31	TX (team* or group*) N3 (educat* or train* or develop* or skill*)
S30	TX (interprofessional or inter professional or inter-professional) N3 (educat* or train* or develop* or skill*)
S29	TX professional development or CPD
S28	(MH "Education, Continuing")
S27	TX skill* N3 develop*
S26	TX educat* or train*
S25	(MH "Health Education")
S24	(MH "Education")
S23	S1 OR S2 OR S3 OR S4 OR S5 OR S6 OR S7 OR S8 OR S9 OR S10 OR S11 OR S12 OR S13 OR S14 OR S15 OR S16 OR S17 OR S18 OR S19 OR S20 OR S21 OR S22
S22	TX (primary care or primary care practice or health care or healthcare or medical care or patient care or care or general practice or family practice) N3 team*
S21	(MH "Multidisciplinary Care Team")
S20	TX health educator*
S19	(MH "Diabetes Educators")
S18	(MH "Health Educators")
S17	TX pharmacist*
S16	(MH "Pharmacists")
S15	TX secretar* or reception* or admin*
S14	(MH "Health Facility Administrators") OR (MH "Clerical Personnel") OR (MH "Administrative Personnel")
S13	TX nurs* or practice nurs* or community nurs* or nurs* practitioner*
S12	(MH "Family Nurse Practitioners")
S11	(MH "Nurse Practitioners")
S10	(MH "Nurses")
S9	TX general practitioner* or medical practitioner* or physician* or clinician* or doctor* or GP*
S8	(MH "Physicians, Family")
S7	(MH "Physicians")
S6	TX (healthcare or health care) N3 (provider* or practitioner* or professional*)
S5	(MH "Medical Staff")
S4	(MH "Health Personnel")
S3	TX primary care or primary medical care or primary health care or primary healthcare or general practice or family practice
S2	(MH "Family Practice")
S1	(MH "Primary Health Care")

ERIC

S81	S16 AND S35 AND S57 AND S72 AND S80
S80	S73 OR S74 OR S75 OR S76 OR S77 OR S78 OR S79
S79	TI group* OR AB group*
S78	TI trial OR AB trial
S77	TI (randomi*ed or randomly) OR AB (randomi*ed or randomly)
S76	TI clinical trial OR AB clinical trial OR SU clinical trial
S75	TI randomi*ed clinical trial OR AB randomi*ed clinical trial OR SU randomi*ed clinical trial
S74	TI controlled clinical trial OR AB controlled clinical trial OR SU controlled clinical trial
S73	TI pragmatic clinical trial OR AB pragmatic clinical trial OR SU pragmatic clinical trial
S72	S58 OR S59 OR S60 OR S61 OR S62 OR S63 OR S64 OR S65 OR S66 OR S67 OR S68 OR S69 OR OR S70 OR S71
S71	TX glucose *tolerance
S70	TX "DM" or "DM1" or "DM2" or "T1D" or "T1DM" or "T2D" or "T2DM" or "NIDDM" or "IDDM" or "MODY"
S69	TX (insulin or noninsulin or non-insulin) N3 (resistan* or depend*)
S68	TX (diabet* or dm) N5 (typ* N3 (two or "2" or II))
S67	TX (diabet* or dm) N5 (typ* N3 (one or "1" or I))
S66	TX diabet*
S65	DE "Diabetes"
S64	TX respiratory sounds
S63	TX bronchial hyperreactivity
S62	TX bronchoconstrict* or bronchospas*
S61	TX bronch* N3 (constrict* or spas*)
S60	TX (bronchial* or respiratory or airway* or lung*) N3 (hypersensitiv* or hyperreactiv* or allerg* or insufficiency)
S59	TX antiasthma* or anti-asthma*
S58	TX asthma or wheez*
S57	S36 OR S37 OR S38 OR S39 OR S40 OR S41 OR S42 OR S43 OR S44 OR S45 OR S46 OR S47 OR S48 OR S49 OR S50 OR S51 OR S52 OR S53 OR S54 OR S55 OR S56
S56	TX glyc*emic control
S55	TX hypoglyc*emia or hyperglyc*emia or diabetic ketoacidosis or hyperglycemic hyperosmolar nonketotic coma or diabetic coma or hyperosmolar hyperglyc*emic nonketotic syndrome or DKA or HNNS or HONK
S54	TX blood glucose self monitoring or HbA1c
S53	DE "Patient Education"
S52	TX (care or service*) N3 (utili*ation or use*)
S51	TX (office or hospital or emergency department or ED or A&E or A & E or "accident and emergency") N3 (visit* or refer* or admission*)
S50	TX out of hours or out-of-hours or OOH
S49	TX hospitali*ation*
S48	TX asthma control test
S47	TX exacerbat* or attack*
S46	TX (self-manag* or self manag* or selfmanag* or self-car* or self car* or selfcar* or self-help or self help or selfhelp or self-administrat* or self administrat* or selfadministrat* or self-monitor* or self monitor* or selfmonitor* or self-medicat* or self medicat* or selfmedicat* or self-treat* or self treat* or selftreat*) N3 plan*
S45	TX (action or treat* or car* or written or manag* or medicat*) N3 plan*
S44	TX patient N3 (relat* or communicat*)
S43	DE "Physician Patient Relationship"
S42	TX quality N3 (care or healthcare or health care)
S41	TX self N3 (manag* or car* or help or administrat* or monitor* or medicat*)
S40	TX self-manag* or selfmanag* or self-car* or selfcar* or self-help or selfhelp or self-administrat* or selfadministrat* or self-monitor* or selfmonitor* or self-medicat* or selfmedicat*
S39	DE "Self Management"
S38	DE "Self Care Skills"
S37	DE "Daily Living Skills"
S36	TX disease management
S35	S17 OR S18 OR S19 OR S20 OR S21 OR S22 OR S23 OR S24 OR S25 OR S26 OR S27 OR S28 OR S29 OR S30 OR S31 OR S32 OR S33 OR S34
S34	TX quality N3 improv*
S33	DE "Nursing Education"
S32	TX continuing medical education or CME
S31	DE "Medical Education"
S30	DE "Staff Development"
S29	DE "Team Training"
S28	DE "Professional Training"
S27	DE "Training"
S26	TX (team* or group*) N3 (educat* or train* or develop* or skill*)
S25	TX (interprofessional or inter professional or inter-professional) N3 (educat* or train* or develop* or skill*)
S24	TX professional development or CPD
S23	DE "Professional Continuing Education"

S22	DE "Continuing Education"
S21	DE "Professional Education"
S20	TX skill* N3 develop*
S19	TX educat* or train*
S18	DE "Health Education"
S17	DE "Education"
S16	S1 OR S2 OR S3 OR S4 OR S5 OR S6 OR S7 OR S8 OR S9 OR S10 OR S11 OR S12 OR S13 OR S14 OR S15
S15	TX (primary care or primary care practice or health care or healthcare or medical care or patient care or care or general practice or family practice) N3 team*
S14	TX health educator*
S13	TX pharmacist*
S12	TX secretar* or reception* or admin*
S11	DE "Administrators"
S10	DE "Clerical Workers"
S9	TX nurs* or practice nurs* or community nurs* or nurs* practitioner*
S8	DE "Nurses"
S7	TX general practitioner* or medical practitioner* or physician* or clinician* or doctor* or GP*
S6	DE "Physicians"
S5	TX (healthcare or health care) N3 (provider* or practitioner* or professional*)
S4	DE "Health Personnel"
S3	TX primary care or primary medical care or primary health care or primary healthcare or general practice or family practice
S2	DE "Family Practice (Medicine)"
S1	DE "Primary Health Care"

Web of Science

# 7	#6<br>*Indexes=SCI-EXPANDED, SSCI, A&HCI, CPCI-S, CPCI-SSH, BKCI-S, BKCI-SSH, ESCI Timespan=1990-2016*
# 6	#5 AND #4 AND #3 AND #2 AND #1<br>*Indexes=SCI-EXPANDED, SSCI, A&HCI, CPCI-S, CPCI-SSH, BKCI-S, BKCI-SSH, ESCI Timespan=All years*
# 5	TS=(clinical trial OR randomized controlled trial OR controlled clinical trial or pragmatic clinical trial)<br>*Indexes=SCI-EXPANDED, SSCI, A&HCI, CPCI-S, CPCI-SSH, BKCI-S, BKCI-SSH, ESCI Timespan=All years*
# 4	TS=(asthma OR wheez* OR "anti asthma*" OR "bronchial* hypersensitiv*" OR "bronchial* hyperreactiv*" OR "bronchial allerg*" OR "bronchial* insufficiency" OR "respiratory hypersensitiv*" OR "respiratory hyperreactiv*" OR "respiratory allerg*" OR "respiratory insufficiency" OR "airway* hypersensitiv*" OR "airway* hyperreactiv*" OR "airway* allerg*" OR "airway* insufficiency" OR "lung* hypersensitiv*" OR "lung* hyperreactiv*" OR "lung allerg*" OR "lung insufficiency" OR "bronch* constrict*" OR "bronch* spas*" OR bronchoconstrict* OR bronchospas* OR "bronchial hyperreactivity" OR "respiratory sounds" OR "diabetes mellitus" OR diabet* OR "type 1 diabetes" OR "type 2 diabetes" OR DM OR DM1 OR DM2 OR T1D OR T1DM OR T2D OR T2DM OR NIDDM OR IDDM OR MODY OR "insulin resistan*" OR "insulin depend*" OR "noninsulin resistan*" OR "noninsulin depend*" OR "non-insulin resistan*" OR "non-insulin depend*" OR "glucose *tolerance")<br>*Indexes=SCI-EXPANDED, SSCI, A&HCI, CPCI-S, CPCI-SSH, BKCI-S, BKCI-SSH, ESCI Timespan=All years*
# 3	TS=(“disease management” OR “self manag*” OR “self car*” OR “self help” OR “self administrat*” OR “self monitor*” OR “self medicat*” OR "qual* care" OR “patient relat*” OR “patient communicat*” OR “self manag* plan*” OR “self car* plan*” OR “self help plan*” OR “self administrat* plan*” OR “self monitor* plan*” OR “self medicat* plan*” OR “action plan*” OR “treat* plan*” OR “car* plan*” OR “written plan*” OR “manag* plan*” OR “medicat* plan*” OR exacerbat* OR attack* OR “asthma control test” OR hospitalization* OR “after hours care” OR “out of hours” OR OOH OR "office visit*" OR "hospital visit*" OR "hospital refer*" OR "hospital admission*" OR "emergency department visit*" OR "emergency department refer*" OR "emergency department admission*" or "ED visit*" OR "ED refer*" OR "ED admission*" OR “A&E visit*" OR “A&E refer*" OR “A&E admission*" OR “accident and emergency visit*" OR “accident and emergency refer*" OR “accident and emergency admission*" OR "care utilization" OR "care use" OR "service utilization" OR "service use" OR “patient education” OR “blood glucose self monitoring” OR HbA1c OR hypoglycemia OR hyperglycemia OR “diabetic ketoacidosis” OR “hyperglycemic hyperosmolar nonketotic coma” OR “diabetic coma” OR “hyperosmolar hyperglycemic nonketotic syndrome” OR DKA OR HNNS OR HONK OR “glycemic control”)<br>*Indexes=SCI-EXPANDED, SSCI, A&HCI, CPCI-S, CPCI-SSH, BKCI-S, BKCI-SSH, ESCI Timespan=All years*
# 2	TS=(educat* OR train* OR skill* OR develop* OR "professional development" OR CPD OR "continuing medical education" OR CME OR "quality improv*")<br>*Indexes=SCI-EXPANDED, SSCI, A&HCI, CPCI-S, CPCI-SSH, BKCI-S, BKCI-SSH, ESCI Timespan=All years*
# 1	TS=("primary care" OR "general practice" OR "family practice" OR "health personnel" OR "healthcare provider*" OR "healthcare practitioner*" OR "healthcare professional*" OR physician* OR "general practitioner*" OR clinician* OR nurs* OR "nurs* practitioner*" OR reception* OR pharmacist* OR "health educator*" OR "care team*" OR "practice team*" OR "general practice team*" OR "family practice team*")<br>*Indexes=SCI-EXPANDED, SSCI, A&HCI, CPCI-S, CPCI-SSH, BKCI-S, BKCI-SSH, ESCI Timespan=All years*

CENTRAL

#1 MeSH descriptor: [Primary Health Care] this term only
#2 MeSH descriptor: [Family Practice] this term only
#3 MeSH descriptor: [General Practice] this term only
#4 primary care or primary medical care or primary health care or primary healthcare or general practice or family practice:ti,ab,kw
#5 MeSH descriptor: [Health Personnel] this term only
#6 MeSH descriptor: [Medical Staff] this term only
#7 (healthcare or health care) near/3 (provider* or practitioner* or professional*):ti,ab,kw
#8 MeSH descriptor: [Physicians] this term only
#9 MeSH descriptor: [Physicians, Primary Care] this term only
#10 MeSH descriptor: [Physicians, Family] this term only
#11 MeSH descriptor: [General Practitioners] this term only
#12 general practitioner* or medical practitioner* or physician* or clinician* or doctor* or GP*:ti,ab,kw
#13 MeSH descriptor: [Nurses] this term only
#14 MeSH descriptor: [Nursing Staff] this term only
#15 MeSH descriptor: [Nurse Practitioners] this term only
#16 MeSH descriptor: [Family Nurse Practitioners] this term only
#17 nurs* or practice nurs* or community nurs* or nurs* practitioner*:ti,ab,kw
#18 MeSH descriptor: [Medical Secretaries] this term only
#19 MeSH descriptor: [Medical Receptionists] this term only
#20 MeSH descriptor: [Health Facility Administrators] this term only
#21 MeSH descriptor: [Administrative Personnel] this term only
#22 secretar* or reception* or admin*:ti,ab,kw
#23 MeSH descriptor: [Pharmacists] this term only
#24 pharmacist*:ti,ab,kw
#25 MeSH descriptor: [Health Educators] this term only
#26 health educator*:ti,ab,kw
#27 MeSH descriptor: [Patient Care Team] this term only
#28 (primary care or primary care practice or health care or healthcare or medical care or patient care or care or general practice or family practice) near/3 team*:ti,ab,kw
#29 #1 or #2 or #3 or #4 or #5 or #6 or #7 or #8 or #9 or #10 or #11 or #12 or #13 or #14 or #15 or #16 or #17 or #18 or #19 or #20 or #21 or #22 or #23 or #24 or #25 or #26 or #27 or #28
#30 MeSH descriptor: [Education] this term only
#31 MeSH descriptor: [Health Education] this term only
#32 educat* or train*:ti,ab,kw
#33 skill* near/3 develop*:ti,ab,kw
#34 MeSH descriptor: [Education, Professional] this term only
#35 MeSH descriptor: [Education, Continuing] this term only
#36 MeSH descriptor: [Staff Development] this term only
#37 professional development or CPD:ti,ab,kw
#38 (interprofessional or inter professional or inter-professional) near/3 (educat* or train* or develop* or skill*):ti,ab,kw
#39 (team* or group*) near/3 (educat* or train* or develop* or skill*):ti,ab,kw
#40 MeSH descriptor: [Education, Medical] this term only
#41 MeSH descriptor: [Education, Medical, Continuing] this term only
#42 continuing medical education or CME:ti,ab,kw
#43 MeSH descriptor: [Education, Nursing] this term only
#44 MeSH descriptor: [Education, Nursing, Continuing] this term only
#45 MeSH descriptor: [Nursing Education Research] this term only
#46 MeSH descriptor: [Nursing Evaluation Research] this term only
#47 MeSH descriptor: [Education, Pharmacy] this term only
#48 MeSH descriptor: [Education, Pharmacy, Continuing] this term only
#49 MeSH descriptor: [Quality Improvement] this term only
#50 quality near/3 improv*:ti,ab,kw
#51 #30 or #31 or #32 or #33 or #34 or #35 or #36 or #37 or #38 or #39 or #40 or #41 or #42 or #43 or #44 or #45 or #46 or #47 or #48 or #49 or #50
#52 MeSH descriptor: [Disease Management] this term only
#53 disease management:ti,ab,kw
#54 MeSH descriptor: [Self Care] this term only
#55 MeSH descriptor: [Self Administration] this term only
#56 MeSH descriptor: [Self Medication] this term only
#57 self-manag* or selfmanag* or self-car* or selfcar* or self-help or selfhelp or self-administrat* or selfadministrat* or self-monitor* or selfmonitor* or self-medicat* or selfmedicat*:ti,ab,kw
#58 self near/3 (manag* or car* or help or administrat* or monitor* or medicat*):ti,ab,kw
#59 MeSH descriptor: [Quality of Health Care] this term only

#60 quality near/3 (care or healthcare or health care):ti,ab,kw
#61 MeSH descriptor: [Professional-Patient Relations] this term only
#62 MeSH descriptor: [Physician-Patient Relations] this term only
#63 MeSH descriptor: [Nurse-Patient Relations] this term only
#64 patient* near/3 (relat* or communicat*):ti,ab,kw
#65 (action or treat* or car* or written or manag* or medicat*) near/3 plan*:ti,ab,kw
#66 (self-manag* or self manag* or selfmanag* or self-car* or self car* or selfcar* or self-help or self help or selfhelp or self-administrat* or self administrat* or selfadministrat* or self-monitor* or self monitor* or selfmonitor* or self-medicat* or self medicat* or selfmedicat* or self-treat* or self treat* or selftreat*) adj3 plan*:ti,ab,kw
#67 exacerbat* or attack*:ti,ab,kw
#68 asthma control test:ti,ab,kw
#69 asthma control test:ti,ab,kw
#70 hospitali*ation*:ti,ab,kw
#71 hospitali*ation:ti,ab,kw
#72 MeSH descriptor: [After-Hours Care] this term only
#73 out of hours or out-of-hours or OOH:ti,ab,kw
#74 (office or hospital or emergency department or ED or A&E or A & E or "accident and emergency") near/3 (visit* or refer* or admission*):ti,ab,kw
#75 (care or service*) near/3 (utili*ation or use*):ti,ab,kw
#76 (care or service*) near/3 (utili*ation or use*):ti,ab,kw
#77 MeSH descriptor: [Patient Education as Topic] this term only
#78 MeSH descriptor: [Blood Glucose Self-Monitoring] this term only
#79 MeSH descriptor: [Hemoglobin A, Glycosylated] this term only
#80 HbA1c:ti,ab,kw
#81 MeSH descriptor: [Hypoglycemia] this term only
#82 MeSH descriptor: [Hyperglycemia] this term only
#83 MeSH descriptor: [Diabetic Ketoacidosis] this term only
#84 MeSH descriptor: [Hyperglycemic Hyperosmolar Nonketotic Coma] this term only
#85 hyperosmolar hyperglyc*emic nonketotic syndrome or DKA or HNNS or HONK:ti,ab,kw
#86 glyc*emic control:ti,ab,kw
#87 #52 or #53 or #54 or #55 or #56 or #57 or #58 or #59 or #60 or #61 or #61 or #63 or #64 or #65 or #66 or #67 or #68 or #69 or #70 or #71 or #72 or #73 or #74 or #75 or #76 or #77 or #78 or #79 or #80 or #81 or #82 or #83 or #84 or #85 or #86
#88 MeSH descriptor: [Asthma] this term only
#89 antiasthma* or anti-asthma*:ti,ab,kw
#90 antiasthma* or anti-asthma*:ti,ab,kw
#91 (bronchial* or respiratory or airway* or lung*) near/3 (hypersensitiv* or hyperreactiv* or allerg* or insufficiency):ti,ab,kw
#92 (bronchial* or respiratory or airway* or lung*) near/3 (hypersensitive* or hyperreactiv* or allerg* or insufficiency):ti,ab,kw
#93 MeSH descriptor: [Bronchial Spasm] this term only
#94 MeSH descriptor: [Bronchoconstriction] this term only
#95 bronchoconstrict* or bronchospas*:ti,ab,kw
#96 bronchial hyperreactivity:ti,ab,kw
#97 respiratory sounds:ti,ab,kw
#98 respiratory sounds:ti,ab,kw
#99 MeSH descriptor: [Diabetes Mellitus] this term only
#100 diabet*:ti,ab,kw
#101 MeSH descriptor: [Diabetes Mellitus, Type 1] this term only
#102 (diabet* or dm) near/5 (typ* near/3 (one or "1" or I)):ti,ab,kw
#103 MeSH descriptor: [Diabetes Mellitus, Type 2] this term only
#104 (diabet* or dm) near/5 (typ* near/3 (two or "2" or II)):ti,ab,kw
#105 (insulin or noninsulin or non-insulin) near/3 (resistan* or depend*):ti,ab,kw
#106 DM or DM1 or DM2 or T1D or T1DM or T2D or T2DM or NIDDM or IDDM or MODY:ti,ab,kw
#107 glucose *tolerance:ti,ab,kw
#108 #88 or #89 or #90 or #91 or #92 or #93 or #94 or #95 or #96 or #97 or #98 or #99 or #100 or #101 or #102 or #103 or #104 or #105 or #106 or #107
#109 MeSH descriptor: [Pragmatic Clinical Trial] this term only
#110 MeSH descriptor: [Clinical Trial] this term only
#111 MeSH descriptor: [Randomized Controlled Trial] this term only
#112 MeSH descriptor: [Controlled Clinical Trial] this term only
#113 controlled clinical trial:pt
#114 clinical trial:pt
#115 pragmatic clinical trial:ti,ab,kw
#116 randomi*ed or randomly:ti,ab
#117 trial:ti,ab
#118 group*:ti,ab
#119 #109 or #110 or #111 or #112 or #113 or #114 or #115 or #116 or #117 or #118
#120 #29 and #51 and #87 and #108 and #119

#121 review or comment or editorial:pt
#122 #120 not #121
#123 MeSH descriptor: [Animals] this term only
#124 #122 not #123
#125 #124 Publication Year from 1990 to 2016, in Trials

BNI

((SU.EXACT("Primary Health Care") OR SU.EXACT("General Practice")) OR (primary care OR primary medical care OR primary health care OR primary healthcare OR general practice OR family practice) OR (SU.EXACT("Staff (Non BNI)") OR SU.EXACT("Medical Profession")) OR (healthcare NEAR/3 (provider* OR practitioner* OR professional*)) OR (health care NEAR/3 (provider* OR practitioner* OR professional*)) OR (general practitioner* OR medical practitioner* OR physician* OR clinician* OR doctor* OR GP*) OR (SU.EXACT("Nurse Practitioner") OR SU.EXACT("Practice Nursing") OR SU.EXACT("Diabetes Nursing")) OR (nurs* OR practice nurs* OR community nurs* OR nurs* practitioner*) OR (secretar* OR reception* OR admin*) OR SU.EXACT("Pharmacists") OR pharmacist* OR (health educator*) OR SU.EXACT("Multidisciplinary Teams") OR (primary care NEAR/3 team*) OR (primary care practice NEAR/3 team*) OR (health care NEAR/3 team*) OR (healthcare NEAR/3 team*) OR (medical care NEAR/3 team*) OR (patient care NEAR/3 team*) OR (care NEAR/3 team*) OR (general practice NEAR/3 team*) OR (family practice NEAR/3 team*)) AND ((educat* OR train*) OR (skill* NEAR/3 develop*) OR (SU.EXACT("Professional Development")) OR (SU.EXACT("Education: Other Professions")) OR (SU.EXACT("Interprofessional Education")) OR (SU.EXACT("Practice Development")) OR (professional development) OR (CPD) OR (interprofessional NEAR/3 (educat* OR train* OR develop* OR skill*)) OR (inter professional NEAR/3 (educat* OR train* OR develop* OR skill*)) OR (inter-professional NEAR/3 (educat* OR train* OR develop* OR skill*)) OR (team* NEAR/3 (educat* OR train* OR develop* OR skill*)) OR (group* NEAR/3 (educat* OR train* OR develop* OR skill*)) OR (continuing medical education) OR (CME) OR (SU.EXACT("Nursing: Education")) OR (SU.EXACT("Community Nursing: Education")) OR (quality NEAR/3 improv*)) AND ((disease management) OR (SU.EXACT("Self Care")) OR (SU.EXACT("Self Medication")) OR (self-manag* OR selfmanag* OR self-car* OR selfcar* OR self-help OR selfhelp OR self-administrat* OR selfadministrat* OR self-monitor* OR selfmonitor* OR self-medicat* OR selfmedicat*) OR (self NEAR/3 (manag* OR car* OR help OR administrat* OR monitor* OR medicat*)) OR (quality NEAR/3 (care OR healthcare OR health care)) OR (SU.EXACT("Nurse Patient Relations")) OR (patient NEAR/3 (relat* OR communicat*)) OR (plan* NEAR/3 (action OR treat* OR car* OR written OR manag* OR medicat*)) OR (plan* NEAR/3 (self-manag* OR "self manag*" OR selfmanag* OR self-car* OR "self car*" OR selfcar* OR self-help OR "self help" OR selfhelp OR self-administrat* OR "self administrat*" OR selfadministrat* OR self-monitor* OR "self monitor*" OR selfmonitor* OR self-medicat* OR "self medicat*" OR selfmedicat* OR self-treat* OR "self treat*" OR selftreat*)) OR (exacerbat* OR attack*) OR (asthma control test) OR (hospitali*ation*) OR (out of hours OR out-of-hours OR OOH) OR (SU.EXACT("Patients: Appointments")) OR (visit* NEAR/3 (office OR hospital OR "emergency department" OR ED OR A E OR "A E" OR "accident and emergency")) OR (refer* NEAR/3 (office OR hospital OR "emergency department" OR ED OR A E OR "A E" OR "accident and emergency")) OR (admission* NEAR/3 (office OR hospital OR "emergency department" OR ED OR A E OR "A E" OR "accident and emergency")) OR (care NEAR/3 (utili*ation OR use*)) OR (service* NEAR/3 (utili*ation OR use*)) OR (SU.EXACT("Patients: Education")) OR (SU.EXACT("Diabetes: Health Promotion")) OR (blood glucose self monitoring) OR (HbA1c) OR (hypoglyc*emia) OR (hyperglyc*emia) OR (diabetic ketoacidosis) OR (hyperglycemic hyperosmolar nonketotic coma) OR (diabetic coma) OR (hyperosmolar hyperglyc*emic nonketotic syndrome OR DKA OR HNNS OR HONK) OR (glyc*emic control)) AND ((SU.EXACT("Asthma")) OR (asthma OR wheez*) OR (antiasthma* OR anti-asthma*) OR ((bronchial* OR respiratory OR airway* OR lung*) NEAR/3 (hypersensitiv* OR hyperreactiv* OR allerg* OR insufficiency)) OR (bronch* NEAR/3 (constrict* OR spas*)) OR (bronchoconstrict* OR bronchospas*) OR (bronchial hyperreactivity) OR (respiratory sounds) OR (SU.EXACT("Diabetes")) OR (diabet*) OR ((diabet* OR dm) NEAR/5 (typ* NEAR/3 (one OR "1" OR I))) OR ((diabet* OR dm) NEAR/5 (typ* NEAR/3 (TWO OR "2" OR II))) OR ((insulin OR noninsulin OR non-insulin) NEAR/3 (resistan* OR depend*)) OR (DM OR DM1 OR DM2 OR T1D OR T1DM OR T2D OR T2DM OR NIDDM OR IDDM OR MODY) OR (glucose intolerance) OR (glucose tolerance)) AND ((pragmatic clinical trial) OR (clinical trial) OR (randomi*ed controlled trial) OR (controlled clinical trial) OR (randomi*ed OR randomly) OR (trial) OR (group*)) AND pd(>19900101)

Additional limits - Date: After 01 January 1990

WHO Global Health Library

(tw:(primary care OR healthcare professional$ OR practice staff OR practice team$)) AND (tw:(educat$ OR train$ OR develop$ OR skill$)) AND (tw:(self-manag$ OR self-monitor$ OR (qual$ NEAR3 care))) AND (tw:(asthma OR diabet$))

In title, abstract, subject

RDRB

a) Advanced search

Search all groups
Keywords: education or training or development
AND
Keywords: asthma or diabetes
Between 1990 and 2016

b) Quick search

Search all groups

Asthma or diabetes

Google Scholar

Advanced search

Find articles

with all of the words: “healthcare professionals” education self-management

with at least one of the words: asthma diabetes

where my words occur: anywhere in the article

Return articles dated between: 1990 — 2016

Unchecked ‘include patents’

Unchecked ‘include citations’

First 500 hits taken
